# Relationship between characteristics of health professionals and the respect for the autonomy of cancer patients at the end of life

**DOI:** 10.1371/journal.pone.0313513

**Published:** 2024-11-12

**Authors:** Júlia Drummond de Camargo, Daniel Neves Forte

**Affiliations:** 1 Palliative nurse on Hospital Samaritano, São Paulo, Brazil; 2 Research and Teaching Institute, Hospital Sírio-Libanês, São Paulo, Brazil; 3 Emergency Department, Central Institute, Hospital das Clínicas da Faculdade de Medicina da Universidade de São Paulo, São Paulo, Brazil; University Medical Centre Ljubljana (UMCL) / Faculty of Medicine, University Ljubljana (FM,UL), SLOVENIA

## Abstract

**Background and aim:**

This study investigated whether providers respected patient’s autonomy, investigating providers’ pattern of decisions and their associated characteristics.

**Methods:**

Cross-sectional study, conducted through anonymous questionnaire with hypothetical clinical cases, presented to providers at one oncology center. Decision-making patterns were pre-stablished accordingly to the response´s pattern.

**Findings:**

Of 151 responses, decisions patterns were paternalistic in 38%, shared in 38%, obstinate in 10.6% and consumerist in 13.2%. The consumerist providers reported never having participated in an EOL class in 35% and 30% had never trained in palliative care. Among providers with paternalistic pattern, 35.1% had never attended ethic lectures. In the obstinate group, 31.2% had no training in palliative care. When asked how subjects saw themselves about their pattern of decision, 100% of obstinate, 95% of consumerist and 89% of paternalistic patterns exhibited cognitive dissonance.

**Conclusion:**

Significative differences between decisions and how the providers judge themselves were observed.

## 1. Introduction

A challenge during end-of-life (EOL) care is avoiding excessive treatment that may cause unnecessary suffering and costs while simultaneously respecting the patient’s autonomy for self-determination at this stage of life. The shared decision model has been shown to be effective in mitigating this challenge. This model is relatively new to professionals and patients, relative to the millenary model of paternalistic decision in which clinicians act unilaterally by making decisions on behalf of patients. The practice of medicine today looks very different than it did 2,500 years ago, though interestingly, there has been greater change in the last 100 years than in the previous 2,400 years. In short, the Hippocratic tradition stands for the proposition that physicians have an ethical obligation to act, to the best of their judgment, for the medical benefit of their patients. Physicians had the responsibility, therefore, to conduct their behavior in such a way as to comply with this obligation [1].

The traditional relationship and communication model between physician and patient have undergone radical changes over the last 40 years, as patients have gained better access to information about health, healthcare, and treatment options. This situation offers an opportunity for a new type of physician-patient partnership, even supported by initiatives to study and understand behavior and shared decision-making in elderly patients [2, 3].

These models are in the middle of a spectrum of decision-making models [4]. Therapeutic obstination is more extreme than paternalism and is defined as the continued and persistent use of measures that sustain the life of patients with advanced diseases, with prolonged maintenance of vital biological systems and delay of death, despite the patients’ preferences and values [5–9]. On the other hand, when decisions are delegated to patients and their families, while the healthcare team only offers the options, it has been called consumerist autonomy. These models that focus exclusively on autonomy may inadvertently compromise patient care by depriving patients and relatives of the professional guidance necessary for making critical decisions at the end of life [10]. Navigating through all these four models of professional-patient relationships (shared, paternalistic, obstinate, and consumerist) reveals essential challenges to the decision-making process.

For patients to participate autonomously in shared decisions, they need to be capable of making choices for themselves about their health care and be aware, competent, and free of undue pressures to make informed decisions at a certain time [11, 12]. This requires professional competency not only to inform patients but also to understand their life values and perceptions about dignity and suffering. Although the alignment of the patients’ biographical values with evidence-based medicine and situational awareness is a technical and difficult issue, it is necessary to ensure that shared decisions are adequate[13, 14].

Also, in Brazil, no law guarantees treatment refusal, nor does a law on EOL care exist. There is a legal rule that a legal representative is responsible for the patient in cases where he is incapacitated. In the absence of law, the best regulation is Resolution 2,232/2019 of the Federal Council of Medicine (CFM), which states that the doctor must respect the patient’s will. However, the recommendation itself makes an exception that in case of a risk to life, the physician must adopt the necessary measures to preserve life, regardless of the patient’s refusal.

Given the complexity of this problem, we hypothesize that knowledge in palliative care and bioethics is associated with a higher frequency of EOL-shared decision-making in oncology. Thus, we aimed to investigate the characteristics of health professionals associated with greater respect for the autonomy of patients with end-stage cancer and analyze their association with the professional’s decision-making pattern.

## 2. Materials and methods

This was a cross-sectional study conducted through an anonymous questionnaire with hypothetical clinical cases based on real-life cases to assess the association between the professionals’ characteristics and their approach to respecting patient autonomy in decision-making during EOL care.

### 2.1. Settings and participants

The eligible participants were health care professionals who agreed to participate by signing the informed consent document in the team of oncology units who work directly with the care of these inpatients (50 nurses, 43 nursing technicians (the professional with training in a technical course, without a graduation course, who performs care at a technical level only), five nutritionists, 14 physiotherapists, three pharmacists and 45 physicians).

The study was conducted in a major hospital located in the metropolitan area of the state of São Paulo, Brazil, with 466 hospitalization beds, including 41 beds for oncology, 31 of them in the oncology hospitalization unit and 10 of them for bone marrow transplantation (BMT).

### 2.2. Development of the questionnaire

After the research ethics committee approved the questionnaire, an initial phase was dedicated to developing it by specialists. Nine specialists from different professions (three nurses, three clinicians, two psychologists, and one nursing technician) with expertise in EOL care were consulted and contributed to improving the questionnaire.

The principal researcher delivered the final version of the informed consent document in person to the professionals.

#### 2.2.1. Creation of scenarios

The following four hypothetical clinical cases formed the basis of the study:

1. Patient with irreversible end-stage cancer, with preserved decision making capacity, choosing to prioritize comfort at end of life and refusing further measures to treat the disease;
2. Patient with irreversible end-stage cancer, with preserved decision making capacity, wishing to receive futile treatments;
3. Patient with non-terminal advanced stage cancer, without information about decision making capacity, presenting an acute reversible illness complication, refusing a potentially beneficial treatment;
4. Patient with non-terminal advanced stage cancer, with decision making capacity, presenting an acute reversible illness complication, refusing a potentially beneficial treatment.

Each question had four answers choices, presented in alternatives from “a” to “d”.

We pre-define all possible combinations of responses choices to the scenarios, categorizing them into pre-specified decision-making profiles:

Consumerist: this provider’s pattern respects patients’ wishes in all scenarios, irrespective of the patient’s decision-making capacity or futility.

Paternalistic: this pattern made decisions without considering the patient’s opinion, believing that the provider knows what will benefit the patient. For instance, in case four, in which the patient refuses a treatment and has decision-making capacity, a paternalistic pattern would not accept the refusal based on patient values. At the same time, this pattern would respect autonomy when there are no more treatment benefits (case 1).

Obstinate: this pattern only respects autonomy when it is according to the provider’s obstination. For instance, the provider would only respect autonomy when patient demands more treatments despite treatment being inappropriate/futile. In the other scenarios, this pattern of answers would not respect patient choices and would choose answers to prolong life irrespective of quality or patient’s values.

Shared-decision: this pattern was defined as those who investigate whether the patient had decision-making capacity before making any decision. We considered the answer to scenario three helpful in differentiating a shared-decision pattern. Thus, in this pattern, providers would respect patient autonomy in case 1. Table 1 shows these patterns in more detail.

**Table 1:** Patterns of decision according to participant’s answers in the scenarios.

	Consumerist	Shared	Paternalistic	Obstinate
Scenario 1 (patient at the end of life, with decision making capacity, and choosing measures of comfort)	Respects	Respects	Respects	Does not respect
Scenario 2 (patient with terminal illness, capable of making decisions, demanding for futile treatments	Respects	Does not respect	Does not respect	Respects
Scenario 3 (patient with advanced cancer and an acute reversible complication, without information about decision-making capacity, who refuses treatment	Respects	Asks for more info	Does not respect	Does not respect
Scenario 4 (patient with advanced cancer and a reversible complication, with decision-making capacity, who refuses further treatments	Respects	Respects	Does not respect	Does not respect

In cases of unforeseen responses that did not fit the above standards, the classification was. previously defined to be based on response priority, as shown below:

- Obstinate: does not respect scenario 1, as this case clearly exemplifies the need for end-of-life care, where invasive interventions would prolong life contrary to patient values.
- Consumerist: respects scenario 2, as this is a case where following the patient’s desire would be compatible with prolonging life with much suffering, not taking into account any technical indication, but only offering what the patient wants without informing him clearly of risks or be responsible for doing something that is not technically indicated.
- Shared: requests more information in scenario 3, as this is a case where it is necessary to understand the patient’s reasons and values for their decision.
- Paternalistic: this does not respect scenario 4, as this is a case in which the patient has decision-making capacity and recognizes the risks of her choice. Not respecting it would be conducted based on the doctor’s values for the patient’s protection and not in agreement with patients values and quality of life.

Furthermore, the participant who asked for more information in the third scenario and did not respect the fourth scenario was considered paternalistic; that participant who asked for more information in the third scenario and respected the fourth scenario was considered shared.

Moreover, the participant who does not respect the 2nd and 3rd scenarios but respected the 4th scenario was considered paternalistic, since he did not ask for more information in the 3rd case to define conduct. We defined this pattern because we believe that this professional would guide his conduct based on what he believes to be best for the patient, regardless of listening to the patient’s opinion.

### 2.3. Data collection and variables

After applying informed consent, the investigator delivered the study questionnaire to the participants in person. The questionnaire was filled out privately to ensure the participants’ anonymity and returned to a sealed urn. Participants received guidance that there would be no right or wrong answer. To avoid bias, they were not introduced to the terms consumerist and obstinate.

Questionnaires were delivered to the professionals during their workdays from July 1, 2020, so they could respond according to their own availability. After one month, we reminded them using a institutional communication chat of the importance of their adherence to the study,. The deadline for questionnaire responses was September 30, 2020.

The answers about the attitudes related to the case were analyzed by comparing the data on the answers about the demographic profile with the justifications given to explain the proposed attitudes in the case.

The following dependent variables were analyzed:

- Index of respect for autonomy, with the professionals’ profile defined according to the extent to which and in which context they respected patient autonomy, as shown in Table 1.
- Presence of cognitive dissonance between the answers and the justifications for the attitudes in the scenarios (variables identified and analyzed post-hoc in the study).

The variable cognitive dissonance was introduced in the study when we realized that there was a contrast between the participants’ answers about the attitudes they deemed correct, their justifications, their profile, and the type of professional they believed to be.

The following independent variables were analyzed:

- Type of professional they believed to be;
- Age;
- Duration of training;
- Time working in oncology;
- Professional training;
- Specialization in oncology and/or palliative care;
- Belief in God or in a higher power.

The S4 File describes the scenarios, answers the participants’ questions, and provides the sociodemographic questionnaire.

### 2.4. Ethics approval and consent to participate

The study was approved by ethics committees at Hospital Sírio Libanês (ID: 2587) and conducted following the Guidelines and Regulatory Norms for Research in Human Beings, in accordance with Resolution N. 466/2012 of the National Health Council and because it is a research with human beings, the Informed Consent Term was applied to all the participants, when the participants were provided written explanations of the survey’s purpose, research methods used, details of participating in and withdrawing from the survey, protection of personal information, and data management methods used in the study.

### 2.5. Statistical analyses

Continuous and semi-continuous variables were compared with a Gauss curve and defined as parametric or non-parametric using the Shapiro-Wilk test. The categorical variables were described as absolute frequency (n) and relative frequency (%) expressed through contingency matrices and analyzed using Pearson’s chi-square test or Fisher’s exact test. The level of significance was set at 5% in all inferential analyses.

Multivariate analysis was performed by multinomial logistic regression, considering the variables with p ≤ 0.50 in the bivariate analysis or the data whose analysis the researchers deemed indispensable as candidate variables. The Hosmer-Lemeshow test was used to evaluate the consistency of the logistic model. Furthermore, probabilities of α ≤ 5% for type I error and β ≤ 20% for type II error were considered.

## 3. Results

Among the 160 oncology team professionals’, we analyzed 151 questionnaires (response rate 94.3%). Of the non-participants, 7 were not at the institution at the time of collection due to vacations or medical / maternity leaves. Two questionnaires were returned blank and were excluded from the analyzes. Professional’s mean age was 35.5 years-old, 111 (73.5%) were women, 63 (48.3%) had graduated over 10 years ago and 51 (33.8%) had worked in oncology over 10 years. According to the pre-established criteria, 57 (37.8%) participants had a paternalistic pattern, 58 (38.4%) shared decisions, 16 (10.6%) were obstinate, and 20 (13.2%) had a consumerist pattern (Tables 2 and 3).

**Table 2:** Profile of answers in the scenarios and characteristics of the participants according to their answer profile (reference group = shared decision-making).

Characteristics	Paternalistic N = 57 (37.8%)	Shared N = 58 (38.4%)	Obstinate N = 16 (10.6%)	Consumerist N = 20 (13.2%)	Significance
**Believes in God or in a higher power—N (%)**					
Yes	53 (93.0%)	54 (93.1%)	14 (87.5%)	19 (95.0%)	P = 0.847
No	4 (7.0%)	4 (6.9%)	2 (12.5%)	1 (5.0%)	
**Age (mean/SD)**	35.71 (± 7.48)	35.46 (± 8.06)	36 (± 8.08)	34.95 (± 4.57)	P = 0.908
**Sex—N (%)**					
Female	44 (77.2%)	38 (65.5%)	13 (81.3%)	16 (80.0%)	P = 0.361
Male	13 (22.8%)	20 (34.5%)	3 (18.7%)	4 (20.0%)	
**Profession—N (%)**					
Nurse	**17 (29.8%)**	14 (24.1%)	**10 (62.5%)**	8 (40.0%)	P < 0.05
Nursing technician	14 (24.6%)	15 (25.9%)	4 (25.0%)	**9 (45.0%)**	
Physiotherapist	6 (10.5%)	6 (10.3%)	0	2 (10.0%)	
Nutritionist	3 (5.3%)	1 (1.7%)	0	1 (5.0%)	
Physician	16 (28.0%)	**20 (34.5%)**	2 (12.5%)	0	
Pharmacist	1 (1.8%)	2 (3.5%)	0	0	
**Training—N (%)**					
Specialization	37 (.0%)	37 (63.7)	9 (56.3%)	7 (35.0%)	P = 0.303
Graduation	14 (24.5%)	4 (6.9%)	3 (18.7%)	5 (25.0%)	
Technical course	1 (1.8%)	15 (25.9%)	4 (25.0%)	7 (35.0%)	
Masters	1 (1.8%)	0	0	1 (5.0%)	
PhD		2 (3.5%)	0	0	
**Duration of training—N (%)**					
Less than 1 years	1 (1.8%)	1 (1.7%)	1 (6.3%)	0	P = 0.412
1 to 3 years	1 (1.8%)	4 (6.9%)	3 (18.7%)	1 (5.0%)	
3 to 5 years	8 (14.0%)	9 (15.5%)	1 (6.3%)	2 (10.0%)	
5 to 10 years	17 (29.8%)	18 (31.0%)	4 (25.0%)	7 (35.0%)	
More than 10 years	**30 (52.6%)**	**26 (44.9%)**	**7 (43.7%)**	**10 (50.0%)**	
**Time working in oncology—N (%)**					
Less than 1 year	1 (1.8%)	3 (5.2%)	1 (6.3%)	1 (5.0%)	P = 0.30
1 to 3 years	7 (12.3%)	13 (22.4%)	3 (18.7%)	2 (10.0%)	
3 to 5 years	11 (19.3%)	14 (24.1%)	2 (12.5%)	3 (15.0%)	
5 to 10 years	18 (31.6%)	11 (19.0%)	4 (25.0%)	6 (30.0%)	
More than 10 years	**20 (35.0%)**	**17 (29.3%)**	**6 (37.5%)**	**8 (40.0%)**	
**Specialization—N (%)**					
Only oncology	**35 (61.4%)**	**38 (65.5%)**	8 (50.0%)	8 (40.0%)	P = 0.181
Only palliative care	0	0	0	0	
Both	3 (5.3%)	4 (6.9%)	0	0	
None	19 (33.3%)	16 (27.6%)	**8 (50.0%)**	**12 (60.0%)**	

*The data with the highest percentages in each category/profile are shown in bold.

*The **nursing technician** is the professional with training in a technical course, without a graduation course, who performs care at a technical level only, such as administering medication, measuring vital signs, performing basic care with patients such as bathing, feeding, hygiene. They are responsible for putting the nursing prescriptions into practice and receive patient care guidelines that are delegated by nurses.

* Statistical analyses used: Chi Square

**Table 3:** Multivariate analysis of the profile of the participants according to the characteristics of the professionals.

Shared X Paternalistic			Shared X Obstinate		Shared X Consumerist	
Characteristics of the participants	OR (CI 95%) OR<1 Favors the shared decision group	P-value	OR (CI 95%) OR<1 Favors the shared decision group	P-value	OR (CI 95%) OR<1 Favors the shared decision group	P-value
Nursing technician	0.33 (0.052–2.13)	0.24	-	-	-	-
Nurse	0.50 (0.084–3.07)	0.46	-	-	-	-
Physician	0.43 (0.06–2.83)	0.38	-	-	-	-
Have oncology specialization	0.51 (0.11–2.27)	0.384	0.39 (0.04–3.17)	0.381	-	-

*P < 0.5 considered in the sample.

* Statistical analyses used: multinomial logistic regression

*OR (Odds ratio): the ratio of the chance that an event will occur in one group to the chance that it will occur in another group.

* The nursing technician had 33% less chance of having a shared decision-making profile; the nurse had a 50% chance of having a shared decision-making or paternalistic profile. and the clinician had 43% less chance of having a shared decision-making profile.

* The professional had 51% less chance of having a shared decision-making profile than having a paternalistic profile if he/she was a specialist in oncology, and 39% less chance of having a shared decision-making profile than having an obstinate profile.

Regarding working time, 30 (52.6%) participants of the paternalistic profile, 10 (50.0%) of the consumerists and 7 (43.7%) of the obstinate ones had graduated for more than 10 years, while in relation to the time of work in oncology, 20 (35.0%), 6 (37.5%) and 8 (40.0%), respectively, from the paternalistic, obstinate and consumerist profiles, had worked for more than 10 years. The analysis of the relationship between the profile of the professionals and the knowledge and experience in palliative and EOL care showed that the number of professionals who had never attended a class on EOL was seven (35.0%) in the consumerist group, 14 (24.6%) in the paternalistic group, three (18.7%) in the obstinate group, and five (8.6%) in the shared decision-making group (p < 0.05) (Tables 4 and 5). In the shared decision-making group, 11 (19.0%) professionals reported never having attended a class on ethics, and five (31.2%) participants in the obstinate group and six (30.0%) participants in the consumerist group stated never having had a course or training in palliative care (p < 0.05).

**Table 4:** Univariate analysis of the profile of the participants according to experience/knowledge in palliative care.

	Paternalistic	Shared	Obstinate	Consumerist	Significance
**Contact with terminal patients**					
Very little	7 (12.3%)	7 (12.1%)	2 (12.5%)	3 (15.0%)	P = 0.498
Little	6 (10.5%)	2 (3.5%)	0	3 (15.0%)	
Regular	18 (31.6%)	12 (20.7%)	2 (12.5%)	3 (15.0%)	
Often	10 (17.5%)	14 (24.1%)	4 (25.0%)	3 (15.0%)	
Very often	16 (28.1%)	23 (39.6%)	8 (50.0%)	8 (40.0%)	
**Class on end-of-life**					
Graduation/technical course	10 (17.5%)	12 (20.7%)	**8 (50.0%)**	**6 (30.0%)**	P < 0.05
Post-graduation	13 (22.8%)	19 (32.8%)	0	3 (15.0%)	
Both	20 (35.1%)	22 (37.9%)	5 (31.3%)	4 (20.0%)	
Never	14 (24.6%)	**5 (8.6%)**	3 (18.7%)	7 (35.0%)	
**Class on ethics**					
Graduation/technical course	10 (17.5%)	15 (25.9%)	**7 (43.8%)**	**8 (40.0%)**	P = 0.142
Post-graduation	16 (28.1%)	20 (34.4%)	2 (12.5%)	4 (20.0%)	
Both	11 (19.3%)	12 (20.7%)	3 (18.7%)	1 (5.0%)	
Never	20 (35.1%)	**11 (19.0%)**	4 (25.0%)	7 (35.0%)	
**Amount of training and courses in palliative care**					
None	11 (19.3%)	10 (17.2%)	**5 (31.2%)**	**6 (30.0%)**	P < 0.05
1	7 (12.3%)	11 (19.0%)	4 (25.0%)	4 (20.0%)	
2 or 3	27 (47.3%)	15 (25.9%)	4 (25.0%)	10 (50.0%)	
4 to 6	3 (5.3%)	9 (15.0%)	1 (6.3%)	0	
More than 6	9 (15.8%)	13 (22.4%)	**2 (12.5%)**	**0**	
**Score attributed to themselves in knowledge in palliative care (mean/SD)**	6.31 (±1.69)	6.8 (±1.14)	6.5(±1.71)	6.3 (±1.08)	P = 0.254

*The data with the highest percentages in each category/profile are shown in bold.

* Statistical analyses used: Chi Square

**Table 5:** Multivariate analysis of the profile of the participants according to knowledge/experience of the professionals (reference group = shared decision-making).

Shared X Paternalistic			Shared X Obstinate		Shared X Consumerist	
Characteristics of the participants	OR (CI 95%) OR<1 Favors the shared decision group	P-value	OR (CI 95%) OR<1 Favors the shared decision group	P-value	OR (CI 95%) OR<1 Favors the shared decision group	P-value
Never attended a class of end-of-life care	10. 38 (1.32–81.11)	0.26	5.96 (0.32–109.88)	0.23	10.87 (0.89–134.34)	0.061
Never attended a class on ethics	-	-	0.14 (0.011–1.76)	0.12	0.28 (0.035–2.33)	0.24
Never received training/course in palliative care	-	-	2.94 (0.17–49.32)	0.45	-	-
Received 1 to 3 training sessions/courses in palliative care	1.87 (0.51–6.77)	0.33	-	-	-	-

*P < 0.5 considered in the sample.

* Statistical analyses used: multinomial logistic regression

*OR (Odds ratio): the ratio of the chance that an event will occur in one group to the chance that it will occur in another group.

Questioned about what type of professional they considered themselves to be regarding EOL care (Table 6), 114 (75.5%) agreed it should be a priority for patients to be able to share their preferences. This justification and self-perception was the same regardless of the professionals’ profile: 100.0% of the participants in the obstinate group, 95.0% in the consumerist group, 89.5% in the paternalistic group, and 25.9% in shared decision-making group presented dissonant justifications for their answers (p < 0.001 in the multivariate analysis—Tables 7 and 8).

**Table 6:** Univariate analysis of the type of professional that the participants considered themselves to be according to their profiles.

You consider yourself a Professional who	Paternalistic	Shared	Obstinate	Consumerist	P-value
Believes that the patient’s preference should be respected	5 (8.8%)	3 (5.2%)	3 (18.7%)	5 (25%)	P < 0.005
Believes that the patient’s preference should be shared	45 (78.9%)	48 (82.7%)	7 (43.8%)	14 (70%)	
Believes that most times the clinician and the health team are responsible for the decision	6 (10.5%)	4 (6.9%)	6 (37.5%)	1 (5%)	
Believes that the patient should have the opportunity to receive treatment at the end of life. until the last second	1 (1.8%)	3 (5.2%)	0	0	

* Statistical analyses used: multinomial logistic regression

*OR (Odds ratio): the ratio of the chance that an event will occur in one group to the chance that it will occur in another group.

**Table 7:** Univariate analysis of the cognitive dissonance of the answers in the scenarios according to the profile of the participants.

Cognitive Dissonance	Paternalistic (57)	Shared (58)	Obstinate (16)	Consumerist (20)	P-value
Yes	51 (89.5%)	15 (25.9%)	16 (100%)	19 (95%)	P < 0.001
No	6 (10.5%)	43 (74.1%)	0	1 (5%)	

* Statistical analyses used: multinomial logistic regression

*OR (Odds ratio): the ratio of the chance that an event will occur in one group to the chance that it will occur in another group.

**Table 8:** Multivariate analysis of the presence of cognitive dissonance between the answers in the scenarios and the profile of the participants according to experience/knowledge in palliative care (reference group = shared decision-making).

Shared X Paternalistic			Shared X Obstinate		Shared X Consumerist	
Cognitive Dissonance	OR (CI 95%) OR<1 Favors the shared decision group	P-value	OR (CI 95%) OR<1 Favors the shared decision group	P-value	OR (CI 95%) OR<1 Favors the shared decision group	P-value
Present	0.011 (0.002–0.074)	0.000	-*	-*	0.0005 (0.000–0.077)	0.000

*P < 0.0001 considered in the sample

* Statistical analyses used: multinomial logistic regression

*OR (Odds ratio): the ratio of the chance that an event will occur in one group to the chance that it will occur in another group.

* Given that 100% of the participants in the “obstinate” group exhibited cognitive dissonance, it was not possible to perform the multivariate test in this group, because it was not possible to multiply the percentage of professionals without cognitive dissonance, whose value was 0.

*The professional had 1.1% less chance of having a shared decision-making profile and 98.9% more chance of having a paternalistic profile when cognitive dissonance was present.

* The professional had 0.5% less chance of having a shared decision-making profile and 99.5% more chance of having a consumerist profile when cognitive dissonance was present.

## 4. Discussion

In the present study there was a high frequency of individuals with paternalistic, obstinate, and consumerist decision-making approaches to the issue, whereas only 38.0% showed a shared-decision approach. Participation in training and courses in palliative care was significantly more frequent among the participants with the latter approach. Remarkably, the justification for respecting patient autonomy was present in 75.0% of the answers regardless of the profile, including in 100.0% of the answers among those with an obstinate decision profile.

### a. Shared decision-making and knowledge of palliative care and bioethics

The relationship between the professionals’ profile and the knowledge and experience in palliative care was analyzed. The results showed that in shared decision-making group, there was a lower percentage of professionals who reported never having an EOL or ethics class, which is in line with the better preparation and knowledge of these professionals.

In Brazil, the data on knowledge and training in palliative care are scarce. One cross-sectional study conducted with students who applied to medical programs at the Federal University of São Paulo showed that the majority felt unprepared for the provision of this type of care, although 50.0% of participants stated having some previous training in palliative care and 73.7% reported having had previous contact with terminal patients [15].

The literature demonstrates divergences and difficulties on the part of the professionals in recognizing the EOL process, which may be associated with lack of experience and knowledge [16]. Most health professionals receive training to treat diseases, with many reporting learning to communicate and discuss approaches to EOL through practice or trial and error, making the process of shared decision-making difficult [17–21].

A randomized clinical trial conducted with oncologists proposed training to help the process of shared decision-making. After the training, these professionals’ approaches significantly improved, with the level of shared decision-making being twice as great as than in other studies in the literature [22, 23].

Curricular content, training, years of experience, and level of education significantly affect the health professionals’ knowledge to be able to provide palliative care, which is reflected in their profile of decision-making [24–29].

The literature shows a relevant association between the professionals’ education, knowledge and EOL care [30]. Many professionals report having learned to communicate with patients and discuss end-of-life decisions through practice and experience, regardless of participation in courses and training. However, clinical experience has emerged as crucial for improving student’s perception of competence and attitude in caring for end-of-life patients. Relying solely on this experience, without a scientific and specific foundation for these practices, may lead professionals to engage in end-of-life discussions only when the patient’s clinical condition is already declining, believing that it is the appropriate conduct and moment, thus hindering the shared decision-making process [19].

These findings align with our study, where we identified that professionals who reported having less experience in training and classes tended to exhibit profiles that did not consider the principles of shared decision-making in end-of-life situations, such as paternalistic, consumerist, and obstinate approaches. This indicates that this type of professional tended to respect patients’ autonomy less.

### b. Self-perception of the professionals, therapeutic obstination and cognitive dissonance

The contradictions in the justifications presented by the professionals led to the *post-hoc* analysis of a new variable, namely *cognitive dissonance*. We observed that dissonance regarding at least one of the assessed scenarios was present in more than half of participants and in all participants with an obstinate profile; the prevalence of dissonance was lower in the shared decision-making group, which demonstrates that the professionals with this profile had their attitudes and cognition were aligned.

The principle of cognitive dissonance was described by the social psychologist Leon Festinger and essentially relates to the fact that people tend to seek consistency in their beliefs and attitudes; a state of conflict occurs when these beliefs are challenged by their actions [31]. Cognitive dissonance is defined as a negative feeling caused by this contradiction. The greater the magnitude of dissonance, the greater the pressure to reduce it, which relates to their ability to adapt and respond to changes, and the extent to which this hinders the decision-making process [32–35].

The findings of the present study are in line with the data in the literature describing that the more serious an error or choice is believed to be, the more people seek to justify their behavior. In the present study, this was reflected in the finding that the individuals with obstinate decisions and attitudes were those who tried more to justify and convince themselves that they had a shared-decision approach toward their patients.

A few years ago, discussing therapeutic obstination was unthinkable because the clinicians were the only stakeholders who had the knowledge, and their decisions were not subjected to any questioning. In the last decades, the previously common obstinate and paternalistic attitudes have being increasingly questioned. This may cause professionals who have worked for many years with a particular opinion on decision-making to be questioned on their practices and face a change that makes them address and understand patients’ rights, such as autonomy in decision-making.

One of the early reviews on the topic was the self-aware interpretation of cognitive dissonance, which is based on the idea that situations creating dissonance occur because there is an inconsistency between an individual’s self-concept and their behavior. In other words, since most of us have a positive self-concept, it is likely that we experience cognitive dissonance when we behave or position ourselves in ways considered incompetent, immoral, or irrational by society or ourselves. Another example is when a doctor chooses among treatment options; they may view their choice as ideal, making it less likely for them to change their mind even if it’s scientifically contested. Furthermore, the desire to reduce dissonance can lead the doctor to become even more committed to the chosen treatment [32–35].

Although some professionals agree that palliative care improves the survival and quality of life, most do not usually recommend it concomitantly with a diagnosis of an advanced or metastatic disease or a disease with a low survival rate. This shows that these professionals appear to give the impression that they are aware of the importance of palliative care (cognition) but, at the same time, offer resistance to its indication (dissonance).

It is inferred that cognitive dissonance causes self-protective distortions in judgments, which may lead not only to errors but also to resistance to acknowledging these errors, including persisting in ineffective treatments [32]. This was found in our study, which examined professionals who exhibied more obstinate attitudes and did not recognize themselves as such.

### c. Limitations

One limitation of our study was the fact that the questionnaire was administered with hypothetical clinical cases through the creation of scenarios. Although the professionals identified and handled the cases according to their clinical experience, they may have depended more on their cognitive and rational capacities rather than the emotional side of decision-making, which may have weighed considerably on the participants’ choices and, therefore, introduced a bias in their answers.

Hypothetical scenarios have limited capacity for generalizability of the findings. This may be accentuated in our study because these professionals who presented cognitive dissonance in their responses to the scenario may have an even greater degree of dissonance when influenced by the emotions associated with decision-making in real life.

Another limitation that we can explain here is that for the analysis of our results, we categorized consumerist and obstinate professionals in a neutral way, also with the intention of avoiding bias in their answers.

The response options are very rigid to reach the purpose of study, we had to be strict in categorizing the participants in the four groups, and we had a probability that some participants thought the researches wanted for answers.

Despite our study’s limitations, we believe that using scenarios for difficult decision-making discussions is a strategy close to the reality experienced daily by professional.

This study was conducted in a single center, which only allowed for analyzing the context of the institution where it was performed. Additionally, the most important limitation of the study was that one of the most relevant parameters of the investigation, the variable cognitive dissonance, was included and analyzed *post-hoc*, which reduced its statistical significance. However, it was shown to be a fundamental issue that warrants further studies.

## 5. Conclusion

Given the hypothesis that greater knowledge in palliative care and bioethics is associated with increased EOL shared decision-making, it was found that the shared decision-making group had the highest percentage of professionals who reported having had a class on EOL and bioethics at some point in their careers. These data demonstrate significant association between the professionals’ participation in training and their decision-making profile, which is probably associated with increased decisions shared with patients.

We believe that this study will benefit future discussions on therapeutic obstination, patient autonomy and, especially, cognitive dissonance with the aim of improving the quality of care provided, as consequence and secondary benefit reducing the financial/therapeutic costs associated with the suffering of patients, families, and health professionals.

## Supporting information

S1 File (PDF)

S2 File (PDF)

S3 File (PDF)

S4 File (DOCX)
