# Supplementary material for: Relationship between characteristics of health professionals and the respect for the autonomy of cancer patients at the end of life
Source: PLoS One. 2024 Nov 12;19(11):e0313513. doi: 10.1371/journal.pone.0313513 (PMC11556740; doi:10.1371/journal.pone.0313513)
Supplement: S1 File — (PDF) [file pone.0313513.s001.pdf]

Questão x Partici	Q1. O que você a	Q2. Qual é o moti	Q3. O que você a	Q4. Qual é o moti	Q5. O que você a
P1	C	D	C	D	B
P2	C	D	C	D	C
P3	C	D	C	C	C
P4	C	D	B	D	C
P5	C	D	C	D	C
P6	C	D	A	D	C
P7	C	D	C	D	C
P8	C	D	C	A	C
P9	B	D	A	D	C
P10	C	C	C	D	C
P11	B	D	A	A	C
P12	C	D	C	D	C
P13	C	D	C	D	C
P14	C	D	C	D	C
P15	C	A	C	D	C
P16	C	D	A	C	C
P17	C	D	C	D	C
P18	C	D	C	D	C
P19	C	D	C	D	C
P20	C	D	C	D	C
P21	C	D	C	D	C
P22	C	D	C	D	C
P23	B	D	C	D	C
P24	C	D	B	B	C
P25	C	D	A	C	C
P26	C	D	B	D	C
P27	C	D	C	D	C
P28	C	C	A	C	B
P29	C	D	C	D	C
P30	C	D	A	D	C
P31	C	D	C	A	C
P32	C	D	C	D	C
P33	B	D	C	D	B
P34	C	D	C	D	A
P35	C	D	C	D	C
P36	B	C	A	C	C
P37	C	D	C	D	C
P38	C	D	A	C	C
P39	C	C	C	D	C
P40	C	D	C	D	C
P41	C	D	C	D	C
P42	B	C	C	D	B
P43	C	D	A	B	C
P44	B	B	C	D	A
P45	C	D	C	D	C
P46	C	D	A	D	C

P47	B	C	B	C	B
P48	C	D	C	C	B
P49	C	C	C	C	C
P50	C	D	C	D	C
P51	C	D	C	D	C
P52	C	D	C	D	C
P53	C	C	C	D	C
P54	C	D	C	D	C
P55	C	D	C	D	C
P56	C	D	C	D	C
P57	C	D	C	D	C
P58	C	D	C	D	C
P59	C	D	A	D	C
P60	C	D	C	D	B
P61	C	D	C	D	C
P62	C	D	C	D	C
P63	C	D	B	B	C
P64	C	D	C	D	C
P65	B	B	C	C	C
P66	C	D	C	D	C
P67	C	D	C	D	C
P68	C	D	C	D	C
P69	C	D	C	D	C
P70	C	D	C	B	C
P71	C	D	B	B	C
P72	C	D	C	D	C
P73	C	D	A	D	C
P74	C	D	C	D	C
P75	C	D	C	D	C
P76	C	D	C	D	C
P77	B	D	B	D	C
P78	C	D	C	D	C
P79	C	D	C	D	C
P80	B	C	B	D	C
P81	B	D	C	D	C
P82	B	C	A	C	B
P83	C	D	B	C	A
P84	C	D	A	D	C
P85	B	B	C	D	C
P86	C	D	C	D	C
P87	C	D	C	D	C
P88	C	D	C	D	C
P89	C	C	C	D	C
P90	C	C	C	C	C
P91	C	D	C	D	B
P92	C	D	C	A	B
P93	C	D	A	D	C

P94	C	D	A	D	C
P95	C	D	C	D	C
P96	C	D	C	D	C
P97	C	D	C	D	C
P98	C	D	A	D	C
P99	C	D	C	D	C
P100	C	D	C	D	C
P101	C	D	C	D	C
P102	C	D	A	D	C
P103	C	D	B	C	C
P104	C	D	C		C
P105	C	D	A	A	B
P106	C	D	B	B	C
P107	C	D	C	D	C
P108	C	D	C	D	C
P109	C	D	B	D	C
P110	C	D	A	C	C
P111	C	D	C	D	C
P112	C	D	C	B	B
P113	C	D	C	D	C
P114	C	D	A	D	C
P115	C	D	C	D	C
P116	C	D	C	D	C
P117	C	D	A	D	C
P118	C	D	C	D	C
P119	C	D	C	D	C
P120	C	D	C	D	C
P121	C	D	C	D	C
P122	C	D	C	D	C
P123	C	D	C	D	C
P124	C	D	C	D	C
P125	C	D	A	C	C
P126	C	D	C	D	C
P127	C	D	C	D	C
P128	C	D	B	D	C
P129	C	D	C	D	C
P130	C	D	C	D	C
P131	C	D	A	C	C
P132	C	D	C	D	A
P133	C	D	C	D	C
P134	C	D	C	D	A
P135	C	D	B	C	A
P136	C	D	C	D	C
P137	C	D	B	B	C
P138	C	D	C	B	C
P139	C	D	C	D	C
P140	C	D	C	D	C

P141	C	D	C	D	C
P142	C	D	C	D	C
P143	C	D	B	B	C
P144	C	D	C	D	A
P145	C	D	C	D	C
P146	C	D	C	D	C
P147	C	D	C	D	C
P148	C	D	C	D	C
P149	B	B	B	D	A
P150	B	D	B	B	A
P151	C	D	B	B	A

Q6. Qual é o moti	Q7. O que você a	Q8. Qual é o moti	Perfil	Dissonancia	Quantidade
C	B	D	Paternalista	Sim	2
D	C	D	Compartilhado	Não	0
D	B	B	Paternalista	Sim	1
D	B	D	Paternalista	Sim	2
B	B	D	Paternalista	Sim	2
D	C	D	Consumerista	Sim	1
D	B	D	Paternalista	Sim	1
D	C	D	Compartilhado	Sim	1
D	B	D	Obstinado	Sim	3
D	C	D	Compartilhado	Sim	1
C	B	D	Obstinado	Sim	3
D	B	D	Paternalista	Sim	1
D	C	D	Compartilhado	Não	0
D	C	D	Compartilhado	Não	0
C	B	D	Paternalista	Sim	3
B	C	C	Consumerista	Sim	1
D	B	D	Paternalista	Sim	1
D	B	D	Paternalista	Sim	1
D	B	D	Paternalista	Sim	1
D	C	D	Compartilhado	Não	0
D	C	D	Compartilhado	Não	0
C	B	C	Paternalista	Sim	2
D	B	D	Obstinado	Sim	2
D	B	D	Paternalista	Sim	1
B	B	D	Consumerista	Sim	2
D	C	D	Compartilhado	Sim	1
D	C	D	Compartilhado	Não	0
C	B	D	Consumerista	Sim	3
D	C	D	Compartilhado	Não	0
B	B	D	Consumerista	Sim	3
B	B	B	Paternalista	Sim	2
D	C	D	Compartilhado	Não	0
D	B	B	Obstinado	Sim	2
C	C	C	**Paternalista**	Não	0
D	C	D	Compartilhado	Não	0
C	B	D	Obstinado	Sim	3
D	C	D	Compartilhado	Não	0
B	C	C	Consumerista	Sim	1
D	C	D	Compartilhado	Sim	1
D	C	D	Compartilhado	Não	0
B	C	D	Compartilhado	Sim	1
C	B	D	Obstinado	Sim	3
D	A	B	Consumerista	Sim	2
B	A	D	Obstinado	Sim	1
D	C	D	Compartilhado	Não	0
D	C	D	Consumerista	Sim	1

C	B	C	Obstinado	Sim	4
D	B	C	Paternalista	Sim	3
D	C	C	Compartilhado	Sim	2
D	C	D	Compartilhado	Não	0
D	C	B	Compartilhado	Sim	1
D	B	D	Paternalista	Sim	1
D	C	D	Compartilhado	Sim	1
C	B	B	Paternalista	Sim	1
D	B	D	Paternalista	Sim	1
D	C	D	Compartilhado	Não	0
D	B	B	Paternalista	Não	0
D	C	D	Compartilhado	Não	0
D	B	D	Consumerista	Sim	2
D	C	D	**Paternalista**	Sim	1
D	C	D	Compartilhado	Não	0
D	B	D	Paternalista	Sim	1
C	B	D	Paternalista	Sim	2
D	B	D	Paternalista	Sim	1
D	B	D	Obstinado	Sim	2
D	B	B	Paternalista	Não	0
D	C	D	Compartilhado	Não	0
D	C	D	Compartilhado	Não	0
D	C	D	Compartilhado	Não	0
C	C	D	Compartilhado	Sim	2
C	B	B	Paternalista	Sim	1
D	C	D	Compartilhado	Não	0
D	B	D	Consumerista	Sim	2
D	B	D	Paternalista	Sim	1
D	C	D	Compartilhado	Não	0
D	C	D	Compartilhado	Não	0
D	B	D	Obstinado	Sim	3
D	C	D	Compartilhado	Não	0
D	C	D	Compartilhado	Não	0
D	B	D	Obstinado	Sim	3
D	C	C	Obstinado	Sim	1
A	C	B	Obstinado	Sim	2
B	B	A	Paternalista	Sim	2
D	B	D	Paternalista	Sim	2
C	B	D	Obstinado	Sim	2
D	B	B	Paternalista	Não	0
D	C	D	Compartilhado	Não	0
B	C	D	Compartilhado	Sim	1
D	C	D	Compartilhado	Sim	1
D	C	C	Compartilhado	Sim	2
D	C	D	**Paternalista**	Sim	1
B	B	B	Paternalista	Sim	2
B	C	C	Consumerista	Sim	2

D	C	D	Consumerista	Sim	1
D	C	D	Compartilhado	Não	0
D	C	D	Compartilhado	Não	0
D	C	D	Compartilhado	Não	0
D	B	D	Consumerista	Sim	2
D	B	D	Paternalista	Sim	1
D	C	D	Compartilhado	Não	0
D	C	D	Compartilhado	Não	0
D	C	D	Consumerista	Sim	1
D	B	D	Paternalista	Sim	2
D	B	D	Paternalista	Sim	1
C	A	D	Consumerista	Sim	2
D	B	B	Paternalista	Não	0
D	B	D	Paternalista	Sim	1
D	C	D	Compartilhado	Não	0
A	B	D	Paternalista	Sim	3
B	B	B	Consumerista	Sim	1
D	B	D	Paternalista	Sim	1
C	B	D	Paternalista	Sim	3
D	B	B	Paternalista	Não	0
D	C	D	Consumerista	Sim	1
D	C	D	Compartilhado	Não	0
D	C	D	Compartilhado	Não	0
D	C	D	Consumerista	Sim	1
D	C	D	Compartilhado	Não	0
B	B	B	Paternalista	Sim	1
D	B	B	Paternalista	Sim	1
D	C	D	Compartilhado	Não	0
D	B	D	Paternalista	Sim	1
D	B	D	Paternalista	Sim	1
D	C	D	Compartilhado	Não	0
D	C	D	Consumerista	Não	0
D	B	D	Paternalista	Sim	1
C	B	D	Paternalista	Sim	2
D	C	D	Compartilhado	Sim	1
D	B	D	Paternalista	Sim	1
D	C	D	Compartilhado	Não	0
B	C	D	Consumerista	Sim	1
D	C	D	**Paternalista**	Sim	1
D	B	D	Paternalista	Sim	1
D	C	D	**Paternalista**	Sim	1
C	B	D	Paternalista	Sim	3
D	A	B	Paternalista	Sim	1
B	B	D	Paternalista	Sim	2
B	C	D	Compartilhado	Sim	2
C	B	B	Paternalista	Sim	1
D	C	D	Compartilhado	Não	0

D	C	D	Compartilhado	Não	0
D	C	D	Compartilhado	Não	0
B	A	D	Paternalista	Sim	2
C	B	D	Paternalista	Sim	1
D	C	D	Compartilhado	Não	0
D	C	D	Compartilhado	Não	0
D	C	D	Compartilhado	Não	0
C	C	D	Compartilhado	Sim	1
C	B	B	Obstinado	Sim	1
C	A	B	Obstinado	Sim	2
C	A	D	Paternalista	Sim	1
			%Paternalista		
			N = 57 (37,8%)		
			%Compartilhado		
			N = 58 (38,4%)		
			%Obstinado		
			N = 16 (10,6%)		
			%Consumerista		
			N = 20 (13,2%)		

Idade	Gênero	Acredita em Deus	Categoria Profissi	Formação:	Tempo de formaç
40	Feminino	Sim	Fisioterapeuta	especialização	5 a 10 anos
30	Feminino	Sim	Farmaceutica	Graduação	5 a 10 anos
33	Feminino	Sim	Enfermeira	especialização	3 a 5 anos
30	Feminino	Sim	Técnico de enferr	Curso técnico	5 a 10 anos
31	Masculino	Sim	Enfermeiro	especialização	Mais de 10 anos
34	Feminino	Sim	Fisioterapeuta	especialização	5 a 10 anos
38	Feminino	Sim	Técnico de enferr	Curso técnico	3 a 5 anos
31	Feminino	Sim	Enfermeiro	Graduação	5 a 10 anos
43	Feminino	Sim	enfermeira	especialização	Mais de 10 anos
29	Feminino	Sim	Enfermeira	especialização	5 a 10 anos
38	Feminino	Sim	Técnico de enferr	Curso técnico	Mais de 10 anos
42	Feminino	Sim	Técnico de enferr	Curso técnico	Mais de 10 anos
32	Masculino	Sim	enfermeiro	especialização	3 a 5 anos
32	Masculino	Sim	Técnico de enferr	curso técnico	1 a 3 anos
37	Feminino	Sim	Técnico de enferr	curso técnico	Mais de 10 anos
43	Masculino	Não	Técnico de enferr	curso de técnico	Mais de 10 anos
26	Feminino	Sim	enfermeiro	especialização	Menos de 1 ano
37	Feminino	Sim	nutricionista	especialização	Mais de 10 anos
28	Masculino	Sim	enfermeiro	graduação	3 a 5 anos
34	Masculino	Sim	médico	especialização	Menos de 1 ano
28	Feminino	Sim	enfermeira	especialização	5 a 10 anos
35	Feminino	Sim	enfermeiro	graduação	Mais de 10 anos
37	Feminino	Sim	enfermeira	especialização	Mais de 10 anos
25	Feminino	Sim	Técnico de enferr	curso técnico	1 a 3 anos
40	Feminino	Sim	enfermeiro	graduação	3 a 5 anos
37	Feminino	Sim	fisioterapeuta	especialização	Mais de 10 anos
29	Feminino	Sim	fisioterapeuta	especialização	3 a 5 anos
35	Feminino	Sim	Técnico de enferr	curso técnico	Mais de 10 anos
39	Feminino	Sim	enfermeiro	especialização	Mais de 10 anos
34	Feminino	Sim	Técnico de enferr	curso técnico	5 a 10 anos
40	Masculino	Sim	Técnico de enferr	curso técnico	Mais de 10 anos
33	Feminino	Sim	fisioterapeuta	graduação	Mais de 10 anos
42	Feminino	Sim	enfermeiro	especialização	Mais de 10 anos
33	Feminino	Sim	Técnico de enferr	curso técnico	5 a 10 anos
28	Feminino	Sim	enfermeiro	especialização	5 a 10 anos
34	Feminino	Sim	enfermeiro	graduação	5 a 10 anos
30	Feminino	Sim	fisioterapeuta	especialização	Mais de 10 anos
39	Feminino	Sim	Técnico de enferr	graduação	Mais de 10 anos
32	Feminino	Sim	Técnico de enferr	curso técnico	Mais de 10 anos
34	Feminino	Sim	Técnico de enferr	curso técnico	1 a 3 anos
36	Feminino	Sim	Técnico de enferr	curso técnico	Mais de 10 anos
40	Feminino	Sim	Técnico de enferr	curso técnico	5 a 10 anos
37	Feminino	Sim	Técnico de enferr	curso técnico	Mais de 10 anos
26	Feminino	Sim	enfermeiro	especialização	3 a 5 anos
39	Feminino	Sim	farmaceutico	especialização	Mais de 10 anos
28	Feminino	Sim	Técnico de enferr	graduação	1 a 3 anos

31	Masculino	Sim	enfermeiro	especialização	Menos de 1 ano
25	Feminino	Não	enfermeiro	especialização	3 a 5 anos
30	Masculino	Sim	Técnico de enferr	curso técnico	Mais de 10 anos
29	Feminino	Sim	Técnico de enferr	curso técnico	5 a 10 anos
59	Feminino	Sim	fisioterapeuta	graduação	Mais de 10 anos
40	Feminino	Sim	enfermeiro	especialização	Mais de 10 anos
29	Feminino	Sim	enfermeiro	especialização	3 a 5 anos
35	Feminino	Sim	Técnico de enferr	curso técnico	5 a 10 anos
34	Masculino	Sim	enfermeiro	graduação	5 a 10 anos
22	Masculino	Sim	Técnico de enferr	curso técnico	3 a 5 anos
37	Feminino	Sim	nutricionista	especialização	Mais de 10 anos
39	Masculino	Sim	Técnico de enferr	curso técnico	Mais de 10 anos
38	Masculino	Sim	enfermeiro	especialização	Mais de 10 anos
30	feminino	Sim	farmaceutico	especialização	5 a 10 anos
29	Feminino	Sim	enfermeiro	especialização	5 a 10 anos
38	Masculino	Sim	fisioterapeuta	doutorado	Mais de 10 anos
29	Feminino	Sim	Técnico de enferr	curso técnico	5 a 10 anos
30	Feminino	Sim	enfermeiro	especialização	5 a 10 anos
30	Feminino	Sim	enfermeiro	especialização	5 a 10 anos
32	Feminino	Sim	fisioterapeuta	mestrado	Mais de 10 anos
24	Feminino	Sim	Técnico de enferr	curso técnico	3 a 5 anos
38	Feminino	Sim	Técnico de enferr	curso técnico	5 a 10 anos
27	Feminino	Sim	nutricionista	especialização	3 a 5 anos
30	Masculino	Sim	enfermeiro	especialização	5 a 10 anos
30	Feminino	Sim	Técnico de enferr	curso técnico	5 a 10 anos
41	Feminino	Sim	enfermeiro	especialização	Mais de 10 anos
27	Feminino	Sim	nutricionista	graduação	5 a 10 anos
42	Feminino	Sim	nutricionista	especialização	Mais de 10 anos
33	Feminino	Sim	Técnico de enferr	curso técnico	Mais de 10 anos
29	Feminino	Sim	fisioterapeuta	especialização	5 a 10 anos
39	Feminino	Não	enfermeiro	graduação	Mais de 10 anos
40	Masculino	Sim	Técnico de enferr	curso técnico	Mais de 10 anos
32	Feminino	Sim	Técnico de enferr	curso técnico	3 a 5 anos
37	Feminino	Sim	Técnico de enferr	curso técnico	5 a 10 anos
26	Feminino	Não	enfermeiro	graduação	1 a 3 anos
27	Feminino	Sim	enfermeiro	especialização	1 a 3 anos
29	Masculino	Sim	Técnico de enferr	curso técnico	5 a 10 anos
30	Feminino	Sim	fisioterapeuta	graduação	5 a 10 anos
28	Feminino	Sim	Técnico de enferr	curso técnico	1 a 3 anos
39	Feminino	Sim	médico	especialização	Mais de 10 anos
39	Feminino	Não	médica	especialização	Mais de 10 anos
58	Feminino	Sim	enfermeira	especialização	Mais de 10 anos
62	Masculino	Sim	médico	doutorado	Mais de 10 anos
42	Masculino	Sim	Técnico de enferr	curso técnico	Mais de 10 anos
37	Feminino	Sim	enfermeira	especialização	Mais de 10 anos
32	Feminino	Sim	Técnico de enferr	curso técnico	3 a 5 anos
30	Feminino	Sim	enfermeira	especialização	5 a 10 anos

31	Feminino	Sim	fisioterapeuta	graduação	5 a 10 anos
30	Feminino	Sim	Técnico de enferr	curso técnico	1 a 3 anos
31	Feminino	Sim	médico	especialização	5 a 10 anos
33	Masculino	Sim	médico	especialização	5 a 10 anos
38	Feminino	Sim	enfermeira	especialização	5 a 10 anos
36	Feminino	Sim	enfermeira	especialização	5 a 10 anos
36	Feminino	Sim	enfermeira	especialização	3 a 5 anos
31	Masculino	Sim	médico	especialização	1 a 3 anos
29	Feminino	Sim	enfermeira	especialização	5 a 10 anos
27	Feminino	Não	fisioterapeuta	especialização	3 a 5 anos
23	Feminino	Sim	Técnico de enferr	curso técnico	3 a 5 anos
30	Masculino	Sim	Técnico de enferr	curso técnico	3 a 5 anos
41	Feminino	Sim	enfermeira	especialização	Mais de 10 anos
33	Feminino	Sim	enfermeira	especialização	Mais de 10 anos
42	Feminino	Sim	médica	especialização	5 a 10 anos
39	Feminino	Não	médica	especialização	Mais de 10 anos
37	Feminino	Sim	enfermeira	especialização	Mais de 10 anos
36	Masculino	Sim	enfermeiro	especialização	Mais de 10 anos
25	Masculino	Sim	Técnico de enferr	curso técnico	5 a 10 anos
35	Feminino	Sim	fisioterapeuta	especialização	Mais de 10 anos
40	Feminino	Sim	enfermeira	mestrado	Mais de 10 anos
37	Masculino	Sim	médico	doutorado	5 a 10 anos
33	Feminino	Sim	médico	especialização	5 a 10 anos
37	Feminino	Sim	enfermeira	especialização	Mais de 10 anos
32	Feminino	Não	médica	especialização	Mais de 10 anos
39	Feminino	Sim	enfermeira	especialização	5 a 10 anos
28	Feminino	Sim	médica	especialização	Mais de 10 anos
34	Feminino	Sim	enfermeira	especialização	Mais de 10 anos
41	Feminino	Sim	enfermeira	especialização	Mais de 10 anos
40	Feminino	Sim	médica	especialização	Mais de 10 anos
42	Masculino	Sim	médico	especialização	Mais de 10 anos
39	Masculino	Sim	Técnico de enferr	curso técnico	Mais de 10 anos
30	Feminino	Sim	enfermeira	especialização	5 a 10 anos
33	Feminino	Sim	médico	especialização	5 a 10 anos
33	Feminino	Sim	médica	especialização	3 a 5 anos
40	Feminino	Sim	médica	especialização	5 a 10 anos
49	Feminino	Sim	enfermeira	especialização	Mais de 10 anos
33	Feminino	Sim	Técnico de enferr	curso técnico	Mais de 10 anos
33	Feminino	Sim	médica	especialização	Mais de 10 anos
52	Masculino	Sim	médico	especialização	Mais de 10 anos
37	Masculino	Sim	médico	especialização	Mais de 10 anos
40	Feminino	Sim	médica	especialização	Mais de 10 anos
40	Feminino	Sim	médica	especialização	Mais de 10 anos
40	Feminino	Sim	médica	especialização	Mais de 10 anos
39	Masculino	Sim	médico	especialização	5 a 10 anos
36	Feminino	Sim	médica	especialização	3 a 5 anos
53	Masculino	Sim	médico	especialização	Mais de 10 anos

37	Feminino	Sim	médica	especialização	Mais de 10 anos
29	Masculino	Não	médico	especialização	5 a 10 anos
63	Masculino	Não	médico	especialização	Mais de 10 anos
58	Masculino	Sim	médico	especialização	Mais de 10 anos
32	Masculino	Sim	médico	especialização	5 a 10 anos
34	Masculino	Sim	médico	especialização	Mais de 10 anos
41	Feminino	Sim	médica	especialização	Mais de 10 anos
44	Masculino	Não	médico	especialização	Mais de 10 anos
56	Masculino	Sim	médico	especialização	Mais de 10 anos
43	Masculino	Sim	médico	especialização	Mais de 10 anos
47	Masculino	Sim	médico	especialização	Mais de 10 anos
Média:	% Feminino	%Sim	%Enf	%especialização	%Menos de 1 ano
35,55629139	N = 111 (73,5%)	N = 140 (92,7%)	N = 49 (32,5%)	N = 90 (59,6%)	N = 3 (2%)
Desvio Padrão:	% Masculino	%Não	%Tec.Enf	%graduação	%1 a 3 anos:
7,415871054	N = 40 (26,5%)	N = 11 (7,3%)	N = 42 (27,8%)	N = 16 (10,6%)	N = 9 (6%)
			%Fisio	%curso tec	%3 a 5 anos:
			N = 14 (9,3%)	N = 40 (26,5%)	N = 20 (13,2%)
			%Nutri	%mestrado	%5 a 10 anos:
			N = 5 (3,3%)	N = 2 (1,3%)	N = 46 (30,5%)
			%Farma	%doutorado	%Mais de 10 anos
			N = 3 (2%)	N = 3 (2%)	N = 73 (48,3%)
			%Médico		
			N = 38 (25,1%)		

Trabalha há quan	Possui especialid	Possui contato co	Já assistiu algum	Já recebeu algum	Já participou de c
1 a 3 anos	Não	Boa – 1 a 2 pacie	Ambas	Na pós graduaçã	Nenhum
Menos de 1 ano	Sim, em oncologi	Muito pouco – 1 a	Na pós graduaçã	Nunca	1
5 a 10 anos	Sim, em ambos	Regular – 1 a 2 pa	Na pós graduaçã	Na pós graduaçã	2 ou 3
5 a 10 anos	Sim, em oncologi	Muito boa – mais	Na graduação/cur	Nunca	4 a 6
Mais de 10 anos	Sim, em oncologi	Regular – 1 a 2 pa	Nunca	Nunca	2 ou 3
3 a 5 anos	Não	Boa – 1 a 2 pacie	Nunca	Nunca	Nenhum
5 a 10 anos	Sim, em oncologi	Muito boa – mais	Ambas	Na graduação/cur	2 ou 3
3 a 5 anos	Sim, em oncologi	Muito boa – mais	Ambas	Na pós graduaçã	1
Mais de 10 anos	Sim, em oncologi	Muito boa – mais	Ambas	Ambas	Mais de 6
5 a 10 anos	Sim, em oncologi	Boa – 1 a 2 pacie	Na pós graduaçã	Na pós graduaçã	1
Mais de 10 anos	Não	Muito pouco – 1 a	Nunca	Nunca	Nenhum
Mais de 10 anos	Não	Muito boa – mais	Nunca	Nunca	Nenhum
3 a 5 anos	Não	Boa – 1 a 2 pacie	Ambas	Ambas	2 ou 3
Mais de 10 anos	Não	Boa – 1 a 2 pacie	Nunca	Nunca	1
5 a 10 anos	Não	Pouco – 1 a 2 pac	Nunca	Nunca	2 ou 3
Mais de 10 anos	Não	Muito boa – mais	Ambas	Nunca	Nenhum
Menos de 1 ano	Sim, em oncologi	Regular – 1 a 2 pa	Na pós graduaçã	Ambas	4 a 6
Mais de 10 anos	Sim, em oncologi	Muito pouco – 1 a	Na pós graduaçã	Na pós graduaçã	2 ou 3
1 a 3 anos	Não	Muito boa – mais	Na graduação/cur	Na graduação/cur	Nenhum
Menos de 1 ano	Sim, em oncologi	Regular – 1 a 2 pa	Na graduação/cur	Na graduação/cur	2 ou 3
5 a 10 anos	Sim, em oncologi	Muito boa – mais	Ambas	Na pós graduaçã	Mais de 6
Mais de 10 anos	Não	Boa – 1 a 2 pacie	Na graduação/cur	Nunca	2 ou 3
Mais de 10 anos	Sim, em oncologi	Muito boa – mais	Ambas	Ambas	4 a 6
5 a 10 anos	Sim, em oncologi	Regular – 1 a 2 pa	Nunca	Nunca	Nenhum
Menos de 1 ano	Não	Regular – 1 a 2 pa	Nunca	Na graduação/cur	Nenhum
5 a 10 anos	Sim, em oncologi	Muito boa – mais	Na pós graduaçã	Na pós graduaçã	2 ou 3
1 a 3 anos	Sim, em oncologi	Boa – 1 a 2 pacie	Ambas	Ambas	Mais de 6
Mais de 10 anos	Não	Muito boa – mais	Na graduação/cur	Na graduação/cur	2 ou 3
3 a 5 anos	Sim, em oncologi	Muito pouco – 1 a	Na pós graduaçã	Na pós graduaçã	4 a 6
5 a 10 anos	Não	Muito boa – mais	Nunca	Nunca	2 ou 3
Mais de 10 anos	Não	Boa – 1 a 2 pacie	Na graduação/cur	Nunca	Mais de 6
1 a 3 anos	Não	Boa – 1 a 2 pacie	Na graduação/cur	Na graduação/cur	Nenhum
5 a 10 anos	Sim, em oncologi	Boa – 1 a 2 pacie	Ambas	Ambas	2 ou 3
5 a 10 anos	Não	Muito boa – mais	Nunca	Nunca	1
1 a 3 anos	Sim, em oncologi	Boa – 1 a 2 pacie	Na pós graduaçã	Na pós graduaçã	2 ou 3
5 a 10 anos	Não	Muito boa – mais	Na graduação/cur	Nunca	Nenhum
5 a 10 anos	Sim, em ambos	Muito boa – mais	Na pós graduaçã	Na pós graduaçã	Mais de 6
5 a 10 anos	Não	Muito boa – mais	Nunca	Na graduação/cur	2 ou 3
5 a 10 anos	Não	Muito boa – mais	Na pós graduaçã	Nunca	Nenhum
Mais de 10 anos	Não	Muito pouco – 1 a	Na graduação/cur	Na graduação/cur	2 ou 3
1 a 3 anos	Não	Pouco – 1 a 2 pac	Nunca	Nunca	1
5 a 10 anos	Não	Muito boa – mais	Nunca	Na graduação/cur	1
Mais de 10 anos	Sim, em oncologi	Muito boa – mais	Nunca	Nunca	2 ou 3
3 a 5 anos	Sim, em oncologi	Muito boa – mais	Ambas	Na pós graduaçã	Nenhum
Mais de 10 anos	Sim, em ambos	Muito boa – mais	Na pós graduaçã	Na pós graduaçã	4 a 6
3 a 5 anos	Não	Muito boa – mais	Na graduação/cur	Na graduação/cur	2 ou 3

Menos de 1 ano	Não	Muito boa – mais	Na graduação/cu	Na graduação/cu		1
3 a 5 anos	Sim, em oncologi	Regular – 1 a 2 p	Na pós graduaçã	Na pós graduaçã	2 ou 3	
Mais de 10 anos	Não	Muito boa – mais	Nunca	Nunca	Nenhum	
1 a 3 anos	Sim, em oncologi	Boa – 1 a 2 pacie	Na pós graduaçã	Na pós graduaçã	2 ou 3	
Mais de 10 anos	Não	Muito boa – mais	Na graduação/cu	Na graduação/cu	Mais de 6	
Mais de 10 anos	Sim, em oncologi	Muito boa – mais	Na pós graduaçã	Nunca	2 ou 3	
1 a 3 anos	Sim, em oncologi	Boa – 1 a 2 pacie	Ambas	Nunca		1
5 a 10 anos	Não	Muito boa – mais	Nunca	Nunca	2 ou 3	
1 a 3 anos	Não	Pouco – 1 a 2 pac	Na graduação/cu	Na graduação/cu		1
3 a 5 anos	Não	Muito boa – mais	Na graduação/cu	Na graduação/cu	2 ou 3	
5 a 10 anos	Não	Regular – 1 a 2 p	Nunca	Ambas	4 a 6	
Mais de 10 anos	Não	Muito boa – mais	Na graduação/cu	Na graduação/cu	Mais de 6	
5 a 10 anos	Sim, em oncologi	Muito boa – mais	Ambas	Na graduação/cu		1
3 a 5 anos	Sim, em ambos	Boa – 1 a 2 pacie	Na pós graduaçã	Na pós graduaçã	2 ou 3	
5 a 10 anos	Sim, em oncologi	Muito boa – mais	Na pós graduaçã	Na pós graduaçã	2 ou 3	
Mais de 10 anos	Não	Muito boa – mais	Ambas	Nunca	2 ou 3	
5 a 10 anos	Não	Muito boa – mais	Nunca	Nunca	Nenhum	
3 a 5 anos	Sim, em oncologi	Boa – 1 a 2 pacie	Ambas	Na graduação/cu	2 ou 3	
5 a 10 anos	Sim, em oncologi	Muito boa – mais	Ambas	Na pós graduaçã	Mais de 6	
5 a 10 anos	Não	Muito boa – mais	Na graduação/cu	Na graduação/cu	Mais de 6	
1 a 3 anos	Não	Boa – 1 a 2 pacie	Na graduação/cu	Na graduação/cu	4 a 6	
3 a 5 anos	Não	Muito boa – mais	Na graduação/cu	Na graduação/cu	Nenhum	
1 a 3 anos	Sim, em oncologi	Muito pouco – 1 a	Ambas	Ambas		1
1 a 3 anos	Não	Muito boa – mais	Ambas	Na pós graduaçã		1
5 a 10 anos	Não	Muito boa – mais	Na graduação/cu	Na graduação/cu	Mais de 6	
Mais de 10 anos	Sim, em oncologi	Muito boa – mais	Ambas	Ambas	2 ou 3	
3 a 5 anos	Não	Boa – 1 a 2 pacie	Na graduação/cu	Nunca	2 ou 3	
5 a 10 anos	Não	Regular – 1 a 2 p	Nunca	Nunca	2 ou 3	
1 a 3 anos	Sim, em oncologi	Regular – 1 a 2 p	Na graduação/cu	Na graduação/cu	4 a 6	
3 a 5 anos	Sim, em oncologi	Muito boa – mais	Ambas	Ambas	4 a 6	
Mais de 10 anos	Não	Muito boa – mais	Nunca	Nunca	2 ou 3	
Mais de 10 anos	Sim, em oncologi	Boa – 1 a 2 pacie	Ambas	Na pós graduaçã	2 ou 3	
3 a 5 anos	Não	Muito pouco – 1 a	Na graduação/cu	Na graduação/cu	2 ou 3	
3 a 5 anos	Não	Boa – 1 a 2 pacie	Na graduação/cu	Na graduação/cu	2 ou 3	
1 a 3 anos	Não	Boa – 1 a 2 pacie	Na graduação/cu	Na graduação/cu		1
1 a 3 anos	Sim, em oncologi	Boa – 1 a 2 pacie	Na graduação/cu	Na graduação/cu	Nenhum	
3 a 5 anos	Sim, em oncologi	Muito pouco – 1 a	Na pós graduaçã	Na graduação/cu	Nenhum	
3 a 5 anos	Não	Regular – 1 a 2 p	Na graduação/cu	Nunca	2 ou 3	
1 a 3 anos	Não	Muito pouco – 1 a	Na graduação/cu	Nunca	2 ou 3	
1 a 3 anos	Sim, em oncologi	Muito boa – mais	Na pós graduaçã	Na pós graduaçã	2 ou 3	
Mais de 10 anos	Sim, em oncologi	Boa – 1 a 2 pacie	Ambas	Ambas	4 a 6	
Mais de 10 anos	Sim, em oncologi	Muito boa – mais	Na pós graduaçã	Na pós graduaçã	Mais de 6	
Mais de 10 anos	Sim, em oncologi	Regular – 1 a 2 p	Na graduação/cu	Nunca	4 a 6	
Mais de 10 anos	Não	Regular – 1 a 2 p	Nunca	Nunca	Nenhum	
Mais de 10 anos	Sim, em oncologi	Muito pouco – 1 a	Ambas	Ambas	2 ou 3	
1 a 3 anos	Não	Regular – 1 a 2 p	Nunca	Nunca	Nenhum	
5 a 10 anos	Sim, em oncologi	Regular – 1 a 2 p	Ambas	Na pós graduaçã	2 ou 3	

5 a 10 anos	Não	Pouco – 1 a 2 pac	Na pós graduaçã	Na pós graduaçã	2 ou 3	
5 a 10 anos	Não	Regular – 1 a 2 p	Na graduação/cu	Na graduação/cu	2 ou 3	
3 a 5 anos	Sim, em oncologi	Muito boa – mais	Ambas	Na pós graduaçã		1
3 a 5 anos	Sim, em oncologi	Boa – 1 a 2 pacie	Ambas	Ambas	2 ou 3	
1 a 3 anos	Sim, em oncologi	Pouco – 1 a 2 pac	Na pós graduaçã	Na pós graduaçã		1
5 a 10 anos	Sim, em oncologi	Pouco – 1 a 2 pac	Na pós graduaçã	Na pós graduaçã	2 ou 3	
Menos de 1 ano	Sim, em oncologi	Regular – 1 a 2 p	Na pós graduaçã	Na graduação/cu		1
1 a 3 anos	Sim, em oncologi	Regular – 1 a 2 p	Ambas	Ambas	Mais de 6	
5 a 10 anos	Sim, em oncologi	Regular – 1 a 2 p	Na pós graduaçã	Na pós graduaçã	2 ou 3	
3 a 5 anos	Sim, em oncologi	Boa – 1 a 2 pacie	Ambas	Na pós graduaçã	Mais de 6	
3 a 5 anos	Não	Muito boa – mais	Nunca	Na graduação/cu	Nenhum	
1 a 3 anos	Não	Boa – 1 a 2 pacie	Na graduação/cu	Na graduação/cu	2 ou 3	
Mais de 10 anos	Sim, em oncologi	Muito boa – mais	Ambas	Ambas	Mais de 6	
Mais de 10 anos	Sim, em oncologi	Regular – 1 a 2 p	Ambas	Na pós graduaçã	Mais de 6	
3 a 5 anos	Sim, em oncologi	Pouco – 1 a 2 pac	Ambas	Ambas	2 ou 3	
1 a 3 anos	Sim, em oncologi	Muito boa – mais	Ambas	Ambas	2 ou 3	
Mais de 10 anos	Sim, em oncologi	Pouco – 1 a 2 pac	Na graduação/cu	Na graduação/cu	Nenhum	
Mais de 10 anos	Sim, em oncologi	Regular – 1 a 2 p	Ambas	Na pós graduaçã		1
3 a 5 anos	Sim, em oncologi	Muito pouco – 1 a	Nunca	Nunca		1
1 a 3 anos	Não	Muito pouco – 1 a	Nunca	Nunca	Nenhum	
Mais de 10 anos	Sim, em oncologi	Muito pouco – 1 a	Na graduação/cu	Na graduação/cu	Nenhum	
5 a 10 anos	Sim, em oncologi	Regular – 1 a 2 p	Ambas	Na graduação/cu	Nenhum	
3 a 5 anos	Sim, em oncologi	Boa – 1 a 2 pacie	Na pós graduaçã	Na pós graduaçã	4 a 6	
Mais de 10 anos	Sim, em oncologi	Muito pouco – 1 a	Ambas	Ambas		1
1 a 3 anos	Sim, em ambos	Muito boa – mais	Ambas	Ambas	Mais de 6	
5 a 10 anos	Sim, em oncologi	Pouco – 1 a 2 pac	Ambas	Na pós graduaçã	2 ou 3	
3 a 5 anos	Sim, em oncologi	Boa – 1 a 2 pacie	Ambas	Na pós graduaçã	Mais de 6	
Mais de 10 anos	Sim, em oncologi	Muito pouco – 1 a	Nunca	Nunca	Mais de 6	
Mais de 10 anos	Sim, em oncologi	Boa – 1 a 2 pacie	Na graduação/cu	Na graduação/cu	2 ou 3	
Mais de 10 anos	Sim, em oncologi	Regular – 1 a 2 p	Ambas	Ambas	2 ou 3	
Mais de 10 anos	Sim, em ambos	Muito boa – mais	Na pós graduaçã	Na pós graduaçã		1
Mais de 10 anos	Não	Muito pouco – 1 a	Nunca	Nunca	Nenhum	
5 a 10 anos	Sim, em oncologi	Regular – 1 a 2 p	Ambas	Ambas	2 ou 3	
5 a 10 anos	Sim, em oncologi	Muito pouco – 1 a	Na pós graduaçã	Nunca	Nenhum	
3 a 5 anos	Sim, em oncologi	Regular – 1 a 2 p	Ambas	Na graduação/cu	Nenhum	
5 a 10 anos	Sim, em oncologi	Regular – 1 a 2 p	Na graduação/cu	Na graduação/cu	2 ou 3	
Mais de 10 anos	Sim, em oncologi	Muito boa – mais	Ambas	Nunca	Mais de 6	
Mais de 10 anos	Não	Muito boa – mais	Nunca	Nunca		1
3 a 5 anos	Sim, em oncologi	Muito boa – mais	Ambas	Ambas	2 ou 3	
Mais de 10 anos	Sim, em oncologi	Regular – 1 a 2 p	Ambas	Nunca	2 ou 3	
Mais de 10 anos	Sim, em oncologi	Pouco – 1 a 2 pac	Ambas	Ambas		1
Mais de 10 anos	Sim, em ambos	Boa – 1 a 2 pacie	Ambas	Na pós graduaçã	Mais de 6	
Mais de 10 anos	Sim, em oncologi	Muito pouco – 1 a	Na pós graduaçã	Na pós graduaçã	2 ou 3	
Mais de 10 anos	Sim, em oncologi	Regular – 1 a 2 p	Ambas	Na pós graduaçã		1
3 a 5 anos	Sim, em oncologi	Muito boa – mais	Na pós graduaçã	Na pós graduaçã	Nenhum	
3 a 5 anos	Sim, em oncologi	Regular – 1 a 2 p	Na pós graduaçã	Ambas		1
Mais de 10 anos	Sim, em oncologi	Regular – 1 a 2 p	Ambas	Nunca	Nenhum	

5 a 10 anos	Sim, em oncologi	Muito boa – mais	Na pós graduaçã	Ambas	2 ou 3	
1 a 3 anos	Sim, em oncologi	Regular – 1 a 2 pa	Ambas	Ambas	4 a 6	
Mais de 10 anos	Sim, em oncologi	Boa – 1 a 2 pacie	Ambas	Ambas	Mais de 6	
Mais de 10 anos	Sim, em oncologi	Regular – 1 a 2 pa	Nunca	Nunca	Nenhum	
3 a 5 anos	Sim, em oncologi	Muito boa – mais	Ambas	Na graduação/cu	Mais de 6	
5 a 10 anos	Sim, em oncologi	Boa – 1 a 2 pacie	Na pós graduaçã	Na pós graduaçã	Mais de 6	
5 a 10 anos	Sim, em oncologi	Muito pouco – 1 a	Na pós graduaçã	Na pós graduaçã	Nenhum	
Mais de 10 anos	Sim, em oncologi	Regular – 1 a 2 pa	Na pós graduaçã	Na pós graduaçã	Mais de 6	
Mais de 10 anos	Sim, em oncologi	Regular – 1 a 2 pa	Na graduação/cu	Na graduação/cu		1
Mais de 10 anos	Sim, em oncologi	Regular – 1 a 2 pa	Na graduação/cu	Na graduação/cu	Nenhum	
5 a 10 anos	Sim, em oncologi	Pouco – 1 a 2 pac	Na pós graduaçã	Na pós graduaçã	2 ou 3	
%Menos de 1 anc	Oncologia:	Muito pouco:	Graduação/curso	Graduação/curso	Nenhum:	
N = 6 (4%)	N = 89 (59%)	N = 19 (12,6%)	36 (23,8%)	N = 40 (26,5%)	N = 32 (21,2%)	
%1 a 3 anos:	Cuidados Paliativ	Pouco:	Pós:	Pós:	1:	
N = 25 (16,5%)	N = 0 (0%)	N = 11 (7,3%)	35 (23,2%)	N = 42 (27,8%)	N = 26 (17,2%)	
%3 a 5 anos:	Ambos:	Regular:	Ambas:	Ambas:	2 ou 3:	
N = 30 (19,9%)	N = 7 (4,6%)	N = 35 (23,2%)	51 (33,8%)	N = 27 (17,9%)	N = 56 (37,1%)	
%5 a 10 anos:	Nenhum:	Boa:	Nunca:	Nunca:	4 a 6:	
N = 39 (25,8%)	N = 55 (36,4%)	N = 31 (20,5%)	29 (19,2%)	N = 42 (27,8%)	N = 13 (8,6%)	
%Mais de 10 anos:		Muito boa:			Mais de 6:	
N = 51(33,8%)		N = 55 (36,4%)			N = 24 (15,9%)	

Nota atribuída a s	Você se considera	Índice de Burnout
6	Acredita que o pa	Risco Baixo
5	Acredita que o pa	Risco Moderado
5	Acredita que o pa	Risco Moderado
7	Acredita que o pa	Risco Baixo
7	Acredita que o pa	Risco Moderado
5	Acredita que o pa	Risco Moderado
7	Acredita que o pa	Risco Moderado
6	Acredita que o pa	Risco Baixo
7	Acredita que na n	Risco Moderado
6	Acredita que o pa	Risco Alto
5	Acredita que o pa	Risco Moderado
7	Acredita que o pa	Risco Moderado
7	Acredita que o pa	Risco Baixo
7	Acredita que o pa	Risco Moderado
5	Acredita que o pa	Risco Moderado
5	Acredita que o pa	Risco Moderado
6	Acredita que o pa	Risco Baixo
7	Acredita que o pa	Risco Baixo
7	Acredita que o pa	Risco Baixo
7	Acredita que o pa	Risco Baixo
6	Acredita que o pa	Risco Moderado
6	Acredita que na n	Risco Baixo
8	Acredita que o pa	Risco Baixo
5	Acredita que o pa	Risco Moderado
5	Acredita que o pa	Risco Baixo
8	Acredita que o pa	Risco Baixo
8	Acredita que o pa	Risco Moderado
6	Acredita que o pa	Risco Baixo
9	Acredita que o pa	Risco Moderado
7	Acredita que na n	Risco Moderado
10	Acredita que o pa	Risco Baixo
6	Acredita que o pa	Risco Moderado
4	Acredita que na n	Risco Moderado
5	Acredita que o pa	Risco Baixo
6	Acredita que o pa	Risco Moderado
3	Acredita que o pa	Risco Moderado
8	Acredita que o pa	Risco Baixo
6	Acredita que o pa	Risco Baixo
5	Acredita que o pa	Risco Baixo
7	Acredita que o pa	Risco Moderado
8	Acredita que o pa	Risco Moderado
8	Acredita que o pa	Risco Moderado
8	Acredita que o pa	Risco Baixo
5	Acredita que o pa	Risco Moderado
8	Acredita que os p	Risco Moderado
8	Acredita que o pa	Risco Moderado

8	Acredita que o pa	Risco Baixo
7	Acredita que o pa	Risco Moderado
6	Acredita que o pa	Risco Baixo
5	Acredita que o pa	Risco Baixo
7	Acredita que na n	Risco Moderado
8	Acredita que o pa	Risco Baixo
6	Acredita que o pa	Risco Alto
7	Acredita que o pa	Risco Moderado
6	Acredita que o pa	Risco Moderado
6	Acredita que o pa	Risco Moderado
5	Acredita que o pa	Risco Moderado
7	Acredita que o pa	Risco Alto
8	Acredita que o pa	Risco Moderado
7	Acredita que na n	Risco Baixo
7	Acredita que o pa	Risco Baixo
6	Acredita que o pa	Risco Moderado
6	Acredita que o pa	Risco Moderado
7	Acredita que o pa	Risco Moderado
7	Acredita que o pa	Risco Baixo
9	Acredita que o pa	Risco Moderado
5	Acredita que o pa	Risco Moderado
8	Acredita que o pa	Risco Baixo
6	Acredita que o pa	Risco Moderado
5	Acredita que o pa	Risco Moderado
7	Acredita que na n	Risco Moderado
6	Acredita que o pa	Risco Moderado
6	Acredita que o pa	Risco Moderado
6	Acredita que o pa	Risco Baixo
8	Acredita que o pa	Risco Alto
5	Acredita que o pa	Risco Baixo
8	Acredita que o pa	Risco Baixo
6	Acredita que na n	Risco Baixo
9	Acredita que os p	Risco Baixo
8	Acredita que o pa	Risco Moderado
7	Acredita que na n	Risco Moderado
4	Acredita que o pa	Risco Moderado
7	Acredita que o pa	Risco Moderado
6	Acredita que o pa	Risco Moderado
7	Acredita que na n	Risco Baixo
5	Acredita que o pa	Risco Baixo
7	Acredita que o pa	Risco Baixo
8	Acredita que o pa	Risco Baixo
7	Acredita que o pa	Risco Baixo
6	Acredita que os p	Risco Moderado
5	Acredita que o pa	Risco Moderado
1	Acredita que o pa	Risco Moderado
6	Acredita que o pa	Risco Moderado

6	Acredita que o pa	Risco Baixo
7	Acredita que o pa	Risco Moderado
7	Acredita que o pa	Risco Moderado
5	Acredita que o pa	Risco Baixo
7	Acredita que o pa	Risco Baixo
2	Acredita que o pa	Risco Moderado
5	Acredita que o pa	Risco Baixo
8	Acredita que o pa	Risco Moderado
8	Acredita que o pa	Risco Moderado
7	Acredita que o pa	Risco Moderado
8	Acredita que na m	Risco Moderado
5	Acredita que o pa	Risco Alto
8	Acredita que o pa	Risco Baixo
7	Acredita que na m	Risco Baixo
7	Acredita que o pa	Risco Moderado
7	Acredita que o pa	Risco Moderado
6	Acredita que o pa	Risco Moderado
7	Acredita que o pa	Risco Moderado
4	Acredita que os p	Risco Baixo
4	Acredita que o pa	Risco Baixo
5	Acredita que o pa	Risco Moderado
7	Acredita que o pa	Risco Baixo
8	Acredita que o pa	Risco Baixo
6	Acredita que o pa	Risco Baixo
8	Acredita que o pa	Risco Moderado
6	Acredita que o pa	Risco Moderado
7	Acredita que o pa	Risco Moderado
8	Acredita que o pa	Risco Moderado
8	Acredita que o pa	Risco Moderado
7	Acredita que o pa	Risco Moderado
8	Acredita que o pa	Risco Baixo
7	Acredita que o pa	Risco Moderado
5	Acredita que o pa	Risco Moderado
5	Acredita que o pa	Risco Moderado
7	Acredita que o pa	Risco Moderado
6	Acredita que o pa	Risco Baixo
7	Acredita que o pa	Risco Baixo
6	Acredita que o pa	Risco Baixo
8	Acredita que o pa	Risco Moderado
7	Acredita que o pa	Risco Baixo
1	Acredita que o pa	Risco Baixo
9	Acredita que o pa	Risco Moderado
7	Acredita que o pa	Risco Moderado
8	Acredita que o pa	Risco Moderado
8	Acredita que na m	Risco Alto
7	Acredita que na m	Risco Baixo
5	Acredita que o pa	Risco Baixo

	8	Acredita que o pa	Risco Moderado
	7	Acredita que o pa	Risco Moderado
	8	Acredita que o pa	Risco Moderado
	6	Acredita que o pa	Risco Moderado
	7	Acredita que o pa	Risco Moderado
	8	Acredita que o pa	Risco Baixo
	5	Acredita que na n	Risco Moderado
	8	Acredita que o pa	Risco Baixo
	8	Acredita que na n	Risco Moderado
	7	Acredita que na n	Risco Moderado
	6	Acredita que o pa	Risco Moderado
Média:		Acredita que o pa	Baixo:
	6,523178808		N = 58 (38,4%)
Desvio Padrão:		Acredita que o pa	Moderado:
	1,436822592		N = 87 (57,6%)
		Acredita que na n	Alto:
			N = 6 (4%)
		Acredita que os pacientes devem ter a oportunidade de receber investimento em fim de vi	

da até o último segundo:
