# Supplementary material for: Relationship between characteristics of health professionals and the respect for the autonomy of cancer patients at the end of life
Source: PLoS One. 2024 Nov 12;19(11):e0313513. doi: 10.1371/journal.pone.0313513 (PMC11556740; doi:10.1371/journal.pone.0313513)
Supplement: S2 File — (PDF) [file pone.0313513.s002.pdf]

Resumo de Teste de Hipótese

	Hipótese nula	Teste	Sig.	Decisão
1	A distribuição de Nota (a si mesmo) dos conhecimentos em cuidados paliativos é a mesma entre as categorias de Q1-Qual a melhor conduta para o médico (Cenário 1)?.	Teste U de Mann-Whitney de amostras independentes	,670	Reter a hipótese nula.

São exibidas significâncias assintóticas. O nível de significância é ,05.

Resumo de Teste de Hipótese

	Hipótese nula	Teste	Sig.	Decisão
1	A distribuição de Nota (a si mesmo) dos conhecimentos em cuidados paliativos é a mesma entre as categorias de Q3-Qual a melhor conduta para o médico (Cenário 2)?.	Teste de Kruskal-Wallis de Amostras Independentes	,004	Rejeitar a hipótese nula.

São exibidas significâncias assintóticas. O nível de significância é ,05.

Amostra 1-Amo...	Estatística Teste
A-B	-42
C-B	26
A-C	-16

Cada linha testa a hipó
Amostra 2 são as mes
São exibidas significân
é ,05.

Descritivos

Q3-Qual a melhor conduta para o médico (Cenário 2)?		A	B	C
Quantidade	Média	1,720	1,895	,757
	Mediana	2,000	2,000	1,000
	Variância	,710	,988	,676
	Desvio Padrão	,8426	,9941	,8223
	Mínimo	0,0	0,0	0,0
	Máximo	3,0	4,0	3,0
	Intervalo	3,0	4,0	3,0
	Intervalo interquartil	1,0	2,0	1,0

Resumo de Teste de Hipótese

	Hipótese nula	Teste	Sig.	Decisão
	A distribuição de Nota (a si mesmo) dos conhecimentos em cuidados	Teste de Kruskal-		[illegible]

1	dos conhecimentos em cuidados paliativos é a mesma entre as categorias de Q5-Qual a melhor conduta para o médico (Cenário 3)?.	Kruskal-Wallis de Amostras Independentes	,085	Reter a hipótese nula.

São exibidas significâncias assintóticas. O nível de significância é ,05.

Resumo de Teste de Hipótese

	Hipótese nula	Teste	Sig.	Decisão
1	A distribuição de Nota (a si mesmo) dos conhecimentos em cuidados paliativos é a mesma entre as categorias de Q7-Qual a melhor conduta para o médico (Cenário 4)?.	Teste de Kruskal-Wallis de Amostras Independentes	,940	Reter a hipótese nula.

São exibidas significâncias assintóticas. O nível de significância é ,05.

de	Std. Erro	Erro Estatística de Teste	Sig.	Sig. Aj.
2,963	12,944	-3,319	,001	,003
[illegible],319	10,588	2,486	,013	,039
[illegible],644	9,448	-1,762	,078	,234

ótese nula de que as distribuições da Amostra 1 e da
smas.
ncias assintóticas (teste de 2 lados). O nível de significância
