# Supplementary material for: Relationship between characteristics of health professionals and the respect for the autonomy of cancer patients at the end of life
Source: PLoS One. 2024 Nov 12;19(11):e0313513. doi: 10.1371/journal.pone.0313513 (PMC11556740; doi:10.1371/journal.pone.0313513)
Supplement: S3 File — (PDF) [file pone.0313513.s003.pdf]

Pseudo R quadrado

Cox e Snell	,657
Nagelkerke	,717
McFadden	,432

Testes de razão de verossimilhança

Efeito	Critérios de ajuste de modelo	Testes de razão de verossimilhança		
	Verossimilhança de log -2 do modelo reduzido	Qui-quadrado	df	Sig.
Interceptação	201,872[a]	0,000	0	
Diss	201,872[a]	0,000	0	
CatProfMod1	218,315	16,443	9	,058
EspOnco2	204,169	2,297	3	,513
TipoProf1	216,189	14,316	9	,112
IndBournout	216,502	14,629	6	,023
Quant1	218,520	16,647	3	,001
AulaCuidPaliat2	207,713	5,840	3	,120
TreinEticaFimVida2	204,859	2,987	3	,394
CursosCuidPaliat3	211,542	9,669	6	,139

A estatística qui-quadrado é a diferença nas verossimilhanças de log -2 entre o [illegible]

a. Esse modelo reduzido é equivalente ao modelo final porque a omissão do efeito [illegible]

Estimativas do parâmetro

Perfil[a]		B	Erro Padrão	Wald	df	Sig.	Exp(B)	Exp[illegible] Limite inferior
Paternalista	Interceptação	-20,632	1,953	111,638	1	,000		
	[Diss=.0]	-4,749	1,040	20,867	1	,000	,009	,001
	[Diss=1.0]	0[b]			0			
	[CatProfMod1=1.0]	-1,014	,959	1,118	1	,290	,363	,055
	[CatProfMod1=2.0]	-,577	,948	,370	1	,543	,562	,088
	[CatProfMod1=3.0]	-,653	,981	,442	1	,506	,521	,076
	[CatProfMod1=4.0]	0[b]			0			
	[EspOnco2=.0]	-,255	,867	,087	1	,768	,775	,142
	[EspOnco2=1.0]	0[b]			0			
	[TipoProf1=1.0]	3,161	2,043	2,393	1	,122	23,586	,430
	[TipoProf1=2.0]	3,906	1,903	4,210	1	,040	49,677	1,191
	[TipoProf1=3.0]	4,106	2,154	3,633	1	,057	60,733	,890
	[TipoProf1=4.0]	0[b]			0			
	[IndBournout=1.0]	19,369	,572	1148,590	1	,000	258218242,220	84237959,198
	[IndBournout=2.0]	19,734	0,000		1		371738978,835	371738978,835
	[IndBournout=3.0]	0[b]			0			
	[Quant1=.00]	0[b]			0			
	[Quant1=1.00]	-1,402	,956	2,150	1	,143	,246	,038
	[Quant1=2.00]	0[b]			0			
	[AulaCuidPaliat2=.0]	2,340	1,049	4,978	1	,026	10,383	1,329
	[AulaCuidPaliat2=1.0]	0[b]			0			

	[TreinEticaFimVida2=.0]	-,449	,822	,299	1	,585	,638	,128
	[TreinEticaFimVida2=1.0]	0[b]			0			
	[CursosCuidPaliat3=.00]	,014	,897	,000	1	,987	1,014	,175
	[CursosCuidPaliat3=1.00]	,628	,656	,918	1	,338	1,874	,518
	[CursosCuidPaliat3=3.00]	0[b]			0			
Obstinado	Interceptação	-56,105	6647,558	,000	1	,993		
	[Diss=.0]	-21,199	2213,959	,000	1	,992	6,212E-10	0,000
	[Diss=1.0]	0[b]			0			
	[CatProfMod1=1.0]	15,660	2576,335	,000	1	,995	6328005,973	0,000
	[CatProfMod1=2.0]	17,362	2576,335	,000	1	,995	34688286,704	0,000
	[CatProfMod1=3.0]	15,827	2576,335	,000	1	,995	7471908,757	0,000
	[CatProfMod1=4.0]	0[b]			0			
	[EspOnco2=.0]	1,264	1,257	1,012	1	,314	3,540	,301
	[EspOnco2=1.0]	0[b]			0			
	[TipoProf1=1.0]	19,367	6128,011	,000	1	,997	257514087,946	0,000
	[TipoProf1=2.0]	19,961	6128,011	,000	1	,997	466633856,571	0,000
	[TipoProf1=3.0]	22,227	6128,011	,000	1	,997	4497931579,549	0,000
	[TipoProf1=4.0]	0[b]			0			
	[IndBournout=1.0]	20,715	,992	436,076	1	,000	992003121,818	141947041,207
	[IndBournout=2.0]	21,343	0,000		1		1858191179,467	1858191179,467
	[IndBournout=3.0]	0[b]			0			
	[Quant1=.00]	0[b]			0			
	[Quant1=1.00]	-4,169	1,270	10,779	1	,001	,015	,001
	[Quant1=2.00]	0[b]			0			
	[AulaCuidPaliat2=.0]	1,786	1,487	1,442	1	,230	5,963	,324
	[AulaCuidPaliat2=1.0]	0[b]			0			
	[TreinEticaFimVida2=.0]	-1,958	1,289	2,308	1	,129	,141	,011
	[TreinEticaFimVida2=1.0]	0[b]			0			
	[CursosCuidPaliat3=.00]	1,078	1,439	,562	1	,454	2,940	,175
	[CursosCuidPaliat3=1.00]	,451	1,186	,145	1	,704	1,570	,154
	[CursosCuidPaliat3=3.00]	0[b]			0			
Consumerista	Interceptação	-37,794	6849,618	,000	1	,996		
	[Diss=.0]	-5,543	1,454	14,531	1	,000	,004	,000
	[Diss=1.0]	0[b]			0			
	[CatProfMod1=1.0]	-,345	1,206	,082	1	,775	,708	,067
	[CatProfMod1=2.0]	-,138	1,209	,013	1	,909	,871	,081
	[CatProfMod1=3.0]	-17,489	2252,101	,000	1	,994	2,539E-08	0,000
	[CatProfMod1=4.0]	0[b]			0			
	[EspOnco2=.0]	,245	1,019	,058	1	,810	1,277	,173
	[EspOnco2=1.0]	0[b]			0			
	[TipoProf1=1.0]	20,735	6474,886	,000	1	,997	1011595126,702	0,000
	[TipoProf1=2.0]	21,089	6474,886	,000	1	,997	1442102118,823	0,000
	[TipoProf1=3.0]	20,829	6474,886	,000	1	,997	1111768393,600	0,000
	[TipoProf1=4.0]	0[b]			0			

	[IndBournout=1.0]	2,033	1,686	1,453	1	,228	7,635	,280
	[IndBournout=2.0]	1,864	1,637	1,296	1	,255	6,450	,261
	[IndBournout=3.0]	0[b]			0			
	[Quant1=.00]	0[b]			0			
	[Quant1=1.00]	-1,860	1,053	3,116	1	,078	,156	,020
	[Quant1=2.00]	0[b]			0			
	[AulaCuidPaliat2=.0]	2,396	1,278	3,515	1	,061	10,978	,897
	[AulaCuidPaliat2=1.0]	0[b]			0			
	[TreinEticaFimVida2=.0]	-1,246	1,068	1,361	1	,243	,288	,035
	[TreinEticaFimVida2=1.0]	0[b]			0			
	[CursosCuidPaliat3=.00]	17,190	2234,530	,000	1	,994	29206718,230	0,000
	[CursosCuidPaliat3=1.00]	17,547	2234,529	,000	1	,994	41750292,136	0,000
	[CursosCuidPaliat3=3.00]	0[b]			0			

a. A categoria de referência é: Compartilhado.

b. Este parâmetro é definido para zero porque é redundante.

c. Um estouro de ponto flutuante ocorreu ao calcular essa estatística. Portanto, seu valor é definido como ausente do sistema.

[illegible](B)
Limite superior
,066
2,376
3,603
3,564
4,237
1293,777
2071,627
4142,967
791527492,469
371738978,835
1,603
81,113

3,195
5,881
6,777
.[c]
.[c]
.[c]
.[c]
41,555
.[c]
.[c]
.[c]
6932657315,934
1858191179,467
,186
109,882
1,765
49,327
16,042
,068
7,524
9,323
.[c]
9,415
.[c]
.[c]
.[c]

208,056
159,680
1,228
134,349
2,334
.[c]
.[c]
