# Supplementary material for: Relationship between characteristics of health professionals and the respect for the autonomy of cancer patients at the end of life
Source: PLoS One. 2024 Nov 12;19(11):e0313513. doi: 10.1371/journal.pone.0313513 (PMC11556740; doi:10.1371/journal.pone.0313513)
Supplement: S4 File — (DOCX) [file pone.0313513.s004.docx]

**Relationship between characteristics of health professionals and the respect for the autonomy of cancer patients at the end of life**

### Supplementary material

**QUESTIONNAIRE - SCENARIOS**

**Creation of the Construct: RELATIONSHIP BETWEEN THE CHARACTERISTICS OF THE PROFESSIONALS IN A HEALTH CARE TEAM AND THE PERCEPTION OF AND RESPECT FOR THE AUTONOMY OF CANCER PATIENTS AT THE END OF LIFE**

**1. Scenario**

**CASE:** A 65 year-old woman with metastatic lung adenocarcinoma, presenting ECOG 4 (cannot carry on self-care and totally confined to bed or chair), is receiving third line of chemotherapy and presenting disease progression (increased lung lesions and new metastases in the central nervous system). She is calm, alert and oriented, and says she is aware of the irreversibility and terminality of her current condition; she talks to her oncologist and relatives, asking not to be given a new line of treatment, preferring to prioritize comfort at the end of life and stay with her family. She mentions preferring that care that is given to her should be aimed at allowing the progression of the disease to a natural and dignified death, because she believes that these conducts would be in accordance with what is most important in her life.

**Q1. What do you believe would be the best conduct of the clinician:**

1. The clinician should try to convince the patient to receive a new line of cancer treatment that would offer a chance, even if small, to increase survival. If the patient does not accept it, the clinician should try to convince the patient’s family that the cancer treatment should be performed. And if the patient progresses with a new clinical complication from the disease, the clinician should convince the patient’s family and move her to the ICU, because this is the only measure that can keep her alive, in case she changes her mind one day.
2. The clinician should try to convince the patient to receive a new line of chemotherapy that would offer a chance, even if small, to increase survival, including a chance of improving the symptoms if there was a response to the treatment. If the patient does not accept it, the clinician should try to convince the patient’s family. Only if the patient suffers a new clinical complication from the disease should the clinician respect the patient’s preference and not move her to the ICU.
3. The clinician should respect the patient’s decision and not give a new line of cancer treatment. The clinician should also indicate in the medical record that the patient is not a candidate for ICU or invasive measures in case of clinical deterioration.

**Q2. What is the justification for your answer to question 1?**

a) I believe that the patient’s life should be preserved at any cost and that all measures available to medicine should be used to this end.

b) I believe that there are situations in which the patients do not know exactly what is best for themselves. In these situations, even if the patient refuses a treatment, it is the clinician’s role to define the best medical conduct, by talking to the patient’s relatives if necessary. After all, it is the clinician’s role to protect the patients from lack of knowledge.

c) I believe that when a patient expresses an opinion, it should be respected, regardless of its benefits or drawbacks.

d) I believe that for an expressed preference to be respected it is necessary that the patient is fully aware of the risks and benefits of his/her choice and that the professional is able to understand the reason for the patient’s choice.

**2. Scenario**

**CASE:** A 34 year-old man is admitted to the oncology inpatient unit due to strong and difficult-to-control pain related to bone metastasis. He had a previous history of spinocellular carcinoma of the paranasal sinus with metastases in the bone and skin and was submitted to three new lines of cancer treatment, the last one with grade 3 toxicity (pneumonitis) that prevented the continuation of the treatment. Currently with ECOG 3 (limited self-care and confined to bed or chair for more than 50% of waking hours), the oncology team determined that there is no further proposal of curative cancer treatment. After adequate pain control and discussion between the team and the patient about prioritizing comfort, without using measures that may cause suffering and allowing the disease to progress to a natural death, the patient asked for all possible measures that may prolong his life to be used, regardless of suffering and quality of life. He also asked to be intubated and taken to the ICU and be kept alive for as long as possible.

**Q3. What do you believe would be the best conduct of the clinician:**

1. The clinician should move the patient to the ICU and use all the necessary invasive measures to keep him alive, according to his preference. This includes intubation, vasoactive drugs, and cardiopulmonary resuscitation if necessary.
2. The clinician should not send the patient to the ICU and should explain to him that, despite his preference, this choice is not compatible with any quality of life beyond the potential prolongation of life.
3. The clinician should try to understand the reason behind the patient’s request. Depending on the motive, transference to the ICU, use of vasoactive drugs at low doses, and orotracheal intubation to begin mechanical ventilation could be indicated but not cardiopulmonary resuscitation, vasoactive drugs at high doses, or hemodialysis.

**Q4. What is the justification for your answer to question 3?**

a) I believe that life should be preserved at any cost, even if it means transference to the ICU, orotracheal intubation with mechanical ventilation, cardiopulmonary resuscitation, and use of high doses of vasoactive drugs; even if the likelihood of recovering is low it may prolong life, as is the patient’s wish.

b) I believe that there are situations in which the medical decision prevails over the patient’s preference because, in this case, moving the patient to the ICU would prolong his life, but without quality, and it is the team’s responsibility to inform and to provide the best care to the patient.

c) I believe the patient’s preference should be respected and followed in all situations, regardless of the benefits or drawbacks.

d) I believe that it is important to talk to the patient to listen and understand the reasons behind his/her preference, as well as to explain any signs and symptoms of clinical deterioration that he/she may be having.

**3. Scenario**

**CASE:** A 76 year-old man diagnosed with extraperitoneal rectal adenocarcinoma, without current active disease, had his last cycle of chemotherapy two months before. He had a rectoprostatic fistula in the late post-operative period (three months) after laparotomy with confection of a Bricker ileal conduit and colostomy. He presented ECOG 1 (restricted in physical activities but ambulatory and able to carry out light work). He was discharged from hospital and then readmitted to the ICU three days later with liver and renal failure that was reversible with dialysis. He complains that living in this way is unbearable and wants to stop dialysis and treatments.

**Q5. What do you believe would be the best conduct of the clinician:**

1. The clinician should maintain dialysis and the remaining treatments necessary for keeping the patient alive for at least 72 hours and then make a reassessment. If the patient does not improve, then stop the treatments and remove the patient from the ICU.
2. The clinician should guide the patient regarding the treatments and the importance of remaining in the ICU at this point and use all possible measures to preserve the patient’s life, and if the patient does not accept, try to convince his family that treatment is necessary.
3. The clinician should talk to the patient and understand the reasons for his request, his life values, and what he understands about his choice, and then inform him about the potential benefits and drawbacks of either maintaining or interrupting the invasive measures. Only then should a decision be made about keeping the patient in the ICU or not.

**Q6. What is the justification for your answer to question 5?**

a) I believe that life should be preserved at any cost even if it means using all measures available to medicine to prolong the patient’s life.

b) I believe that the patient’s preference should be respected in all situations and that this means quality of life for him.

c) I believe that there are situations in which the medical decision prevails over the patients’ preference because professionals have the knowledge to decide and enforce what is best for the patient and may cure him/her, and that the professionals should talk to the patient’s family if necessary. After all, it is the clinician’s role to protect the patients from lack of knowledge.

d) I believe that the patient should receive all the information about the prognosis of the disease and be allowed to discuss his/her values and the concept of quality of life, being fully aware of the risks and benefits of his/her choice, thus making known the reasons for his/her choice.

**4. Scenario**

**CASE:** A 26 year-old woman is diagnosed with non-small cell lung cancer with metastases in the bone and central nervous system, without neurological symptoms, and is presenting ECOG 2 (unable to carry out any work activities but ambulatory and capable of self-care). She was informed about the first line of treatment and refused it, even after the team made it clear that not being treated could worsen the symptoms with the progression of the disease and her survival would be significantly shorter than if she had the treatment. Moreover, the clinician informed the patient (and she understood) that a good percentage of patients had fewer symptoms and lived longer after receiving the treatment, compared with patients who were not treated and developed more symptoms and lived for a lesser period of time. Nevertheless, the patient stated that she would not want to receive the treatments or come to the hospital, as that would prevent her from living her life the way she wants, even if that meant having more symptoms and a shorter survival.

**Q7. What do you believe would be the best conduct of the clinician:**

1. In this case, it is unacceptable that the patient does not receive treatment. The clinician should be emphatic in the opinion that not undergoing treatment is out of the question because the patient has metastases and the treatment may be beneficial to control the symptoms and prolong life. If the patient still refuses the treatment, the clinician should talk to her family to convince her to use all the treatments validated for this type of cancer, including radiotherapy and, eventually, a second or third line of treatment in case of initial deterioration.
2. The clinician should convince the patient that it is necessary to receive the first line of treatment and only discuss the patient’s preferences if there is no response to the treatment and the disease progresses.
3. The clinician should respect the patient’s preference of not receiving the indicated first line of cancer treatment.

**Q8. What is the justification for your answer to question 7?**

1. I believe that life should be preserved at any cost, especially when there is a chance of cure or stabilization of the disease, even if it means insisting on several lines of treatment and using invasive measures to sustain life, if necessary.
2. I believe that there are situations in which the principles of quality of life should guide medical decisions and that these prevail over the patient’s preferences because, in this case, not receiving the cancer treatment would mean allowing the disease to progress and it is the team’s responsibility to inform and ensure the best care for the patient.
3. I believe that the patient’s preference should be respected in any situation.
4. The patient should receive all the information about the prognosis of the disease and the symptoms that may appear with the clinical worsening of the disease; then the clinician should talk again with the patient in due time to understand the reasons for her preference and inform her of the risks of her decision, and respect it.

**Q9. What other important issue do you think should be discussed?**

**QUESTIONNAIRE – CHARACTERIZATION**

The aim of these questions is to characterize the profile of the healthcare professionals working in the oncology unit of the Sírio-Libanês Hospital. The questionnaire is anonymous and your HONEST answer is very valuable.

Please be honest, this questionnaire is anonymous and we will use this information to improve our work.

**Characterization**

- **Age:**
- **Sex:**

Female ( )

Male ( )

Non-binary ( )

- **Do you believe in God or in a higher power?**

Yes ( )

No ( )

- **Professional category:**
- **Training:**
- **Duration of training:**

Less than 1 year ( )

1 to 3 years ( )

3 to 5 years ( )

5 to 10 Years ( )

More than 10 years ( )

- **How long have you worked in oncology:**

Less than 1 year ( )

1 to 3 years ( )

3 to 5 years ( )

5 to 10 years ( )

More than 10 years ( )

- **Are you a specialist in oncology and/or palliative care**

Yes, in oncology ( )

Yes, in palliative care ( )

Yes, in both

No ( )

- **Does your work involve contact with patients in palliative care and at the end of life?**

Very little – 1 to 2 patients per month ( )

Little – 1 to 2 patients every 15 days ( )

Regular – 1 to 2 patients per week ( )

Often – 1 to 2 patients per day ( )

Very often – more than 2 patients per day ( )

- **Have you attended an end-of-life or palliative care class?**

During graduation ( )

During post-graduation ( )

Both ( )

Never ( )

- **Have you received any training or studied about end-of-life legislation and/or ethics?**

During graduation ( )

During post-graduation ( )

Both ( )

Never ( )

- **Have you attended courses, conferences, or lectures related to palliative care?**

None ( )

1 ( )

2 or 3 ( )

4 to 6 ( )

More than 6 ( )

- **What score can you give yourself on knowledge about palliative care (zero to ten):**
- **You consider yourself a professional who:**

( ) Believes that the patient’s preference should be respected, regardless of the consequences, because you think that this means respecting patient autonomy.

( ) Believes that the patient’s preference should be shared, discussed, and understood to respect his/her wishes, because you think this means respecting patient autonomy.

( ) Believes that, most of the times, the clinician and the healthcare team are responsible for guiding the patient’s decision, because the professional has more knowledge about the consequences that the patient may suffer as a result of his/her choices.

( ) Believes that patients should have the opportunity to receive investment at the end of life, until the last second, because everyone has the right to “fight” and have hope.
